# Intergenomic gene transfer in diploid and allopolyploid *Gossypium*

**DOI:** 10.1186/s12870-019-2041-2

**Published:** 2019-11-12

**Authors:** Nan Zhao, Corrinne E. Grover, Zhiwen Chen, Jonathan F. Wendel, Jinping Hua

**Affiliations:** 1 Laboratory of Cotton Genetics, Genomics and Breeding /Joint Laboratory for International Cooperation in Crop Molecular Breeding, Ministry of Education / Key Laboratory of Crop Heterosis and Utilization of Ministry of Education, College of Agronomy and Biotechnology, China Agricultural University, Beijing, 100193 China; 2 Department of Ecology, Evolution and Organismal Biology, Iowa State University, Ames, IA 50011 USA

**Keywords:** Intergenomic gene transfer, Allopolyploidization, *Gossypium*, Mitochondrial genome, Chloroplast genome, *Numt*, *Nupt*

## Abstract

**Background:**

Intergenomic gene transfer (IGT) between nuclear and organellar genomes is a common phenomenon during plant evolution. *Gossypium* is a useful model to evaluate the genomic consequences of IGT for both diploid and polyploid species. Here, we explore IGT among nuclear, mitochondrial, and plastid genomes of four cotton species, including two allopolyploids and their model diploid progenitors (genome donors, *G. arboreum*: A_2_ and *G. raimondii*: D_5_).

**Results:**

Extensive IGT events exist for both diploid and allotetraploid cotton (*Gossypium*) species, with the nuclear genome being the predominant recipient of transferred DNA followed by the mitochondrial genome. The nuclear genome has integrated 100 times more foreign sequences than the mitochondrial genome has in total length. In the nucleus, the integrated length of chloroplast DNA (cpDNA) was between 1.87 times (in diploids) to nearly four times (in allopolyploids) greater than that of mitochondrial DNA (mtDNA). In the mitochondrion, the length of nuclear DNA (nuDNA) was typically three times than that of cpDNA. *Gossypium* mitochondrial genomes integrated three nuclear retrotransposons and eight chloroplast tRNA genes, and incorporated chloroplast DNA prior to divergence between the diploids and allopolyploid formation. For mitochondrial chloroplast-tRNA genes, there were 2-6 bp conserved microhomologies flanking their insertion sites across distantly related genera, which increased to 10 bp microhomologies for the four cotton species studied. For organellar DNA sequences, there are source hotspots, e.g., the *atp6*-*trnW* intergenic region in the mitochondrion and the inverted repeat region in the chloroplast. Organellar DNAs in the nucleus were rarely expressed, and at low levels. Surprisingly, there was asymmetry in the survivorship of ancestral insertions following allopolyploidy, with most *numts* (nuclear mitochondrial insertions) decaying or being lost whereas most *nupts* (nuclear plastidial insertions) were retained.

**Conclusions:**

This study characterized and compared intracellular transfer among nuclear and organellar genomes within two cultivated allopolyploids and their ancestral diploid cotton species. A striking asymmetry in the fate of IGTs in allopolyploid cotton was discovered, with *numts* being preferentially lost relative to *nupts.* Our results connect intergenomic gene transfer with allotetraploidy and provide new insight into intracellular genome evolution.

## Background

Prokaryotic α-proteobacteria and cyanobacteria are known to be the forerunners of modern eukaryotic mitochondria [1] and chloroplasts [2, 3], as described by the endosymbiont theory. The transformation from endosymbionts to organelles was accompanied by massive DNA transfer among intracellular genomes, or intergenomic gene transfers (IGT). Although the pace of IGT has slowed considerably since eukaryote formation, it remains a common process that is characteristic of nuclear and organellar genome evolution in plants [4]. Among the three types of genomes in a plant cell, there are six possible directions of gene transfer. The most prominent directions of IGT are from either organellar genome into the nuclear genome [2, 4–25], then from the nuclear and plastid genomes into the mitochondrial genomes [2, 26–38]. Interorganellar transfer to the highly compact plastid genome appears to be quite rare [39–43] .

Recent research has revealed that plant mitochondrial genomes frequently integrate DNA from the other two cellular compartments. As the number of sequenced plant mitochondrial genomes has increased [27, 29, 39, 42, 44–61], the extent of integration from both the chloroplast [28, 30, 32, 62, 63] and nuclear genomes [33, 60, 62] has become more apparent. In general, plant mitochondrial genomes have between 0.56% (*Marchantia polymorpha*) – 10.85% (*Phoenix dactylifera*) plastid-derived sequences [9, 10, 34]. Nuclear sequence integration tends to be more abundant and more difficult to identify, as these commonly include retrotransposon and other repetitive fragments [28, 31, 33, 55].

Nuclear integrants derived from the mitochondrial and plastic genomes are termed *numts* [19] and *nupts* [4], respectively, with these collectively referred to as norgDNAs [14], or nuclear organellar DNAs. Environmental stresses have been shown to increase entry of organellar DNA into the nucleus in plants [8], with insertions commonly occurring in open chromatin regions [12]. While norgDNAs are commonly thought to be inactive, there is some evidence of norgDNA transcription in plant species, including in rice [11] and cotton [64].

While IGT has relevance to broad questions of genome evolution, IGT takes on additional importance due to its possible relationship to plant fertility [65–68]. Repeated IGT transfers can create regions of homology within the mitochondrial genome, which can provide hotspots for intra-organellar recombination. Mitogenomic recombination is a common phenomenon that may generate novel chimeric sequences [69–72]. These novel chimeric sequences may co-transcribe with adjacent functional genes [73–75], subsequently affecting or interfering with mitochondrial electron transfer chain pathways [76, 77]. Furthermore, the phenomenon known as cytoplasmic male sterility is influenced both by the nuclear and mitochondrial compartments, as well as by their interactions [65]. Thus, improving our understanding of IGT may help inform breeding strategies that utilize plant fertility differences, e.g., in male sterility to develop hybrids.

The genus *Gossypium* contains approximately 50 species [78–80], including four that have been domesticated and presently are cultivated for their seed trichomes, or cotton fiber. Two of these domesticated species belong to the clade of seven extant allopolyploid species (AD genome), which formed about 1–2 million years ago (Mya) when an A-genome diploid (resembling modern *G. arboreum*, A_2_ or *G. herbaceum,* A_1_) hybridized with a D-genome species (resembling modern *G. raimondii*, D_5_) and subsequently doubled in chromosome number [79, 81]. High-quality nuclear genome assemblies have recently become available for the diploids *G. raimondii* [64, 82] and *G. arboreum* [83], and for the allopolyploids *G. hirsutum* [84–86] and *G. barbadense* [86–88]. There also exist multiple organellar genome sequences for both diploid and allopolyploid cotton species [60, 61, 63, 65, 89–93]. These genomic data provide the foundation for discovery and description of IGT events in *Gossypium*.

Here we analyze intracellular transfer among nuclear and organellar genomes within four cotton species, including the two cultivated allopolyploids (AD genome) and models of their ancestral diploid (A, D) genome donors, to explore the prevalence of IGT in cotton (*Gossypium*). We characterize and compare the frequencies of the six possible classes of IGT events, as well as the sources and sizes of the inter-organellar sequences. We report a striking asymmetry in the fate of IGTs in allopolyploid cotton, with *numts* being preferentially lost relative to *nupts.* Finally, we explored the expression of norgDNAs.

## Results

### General profiles of intergenomic gene transfer in *Gossypium*

**Fig. 1:**
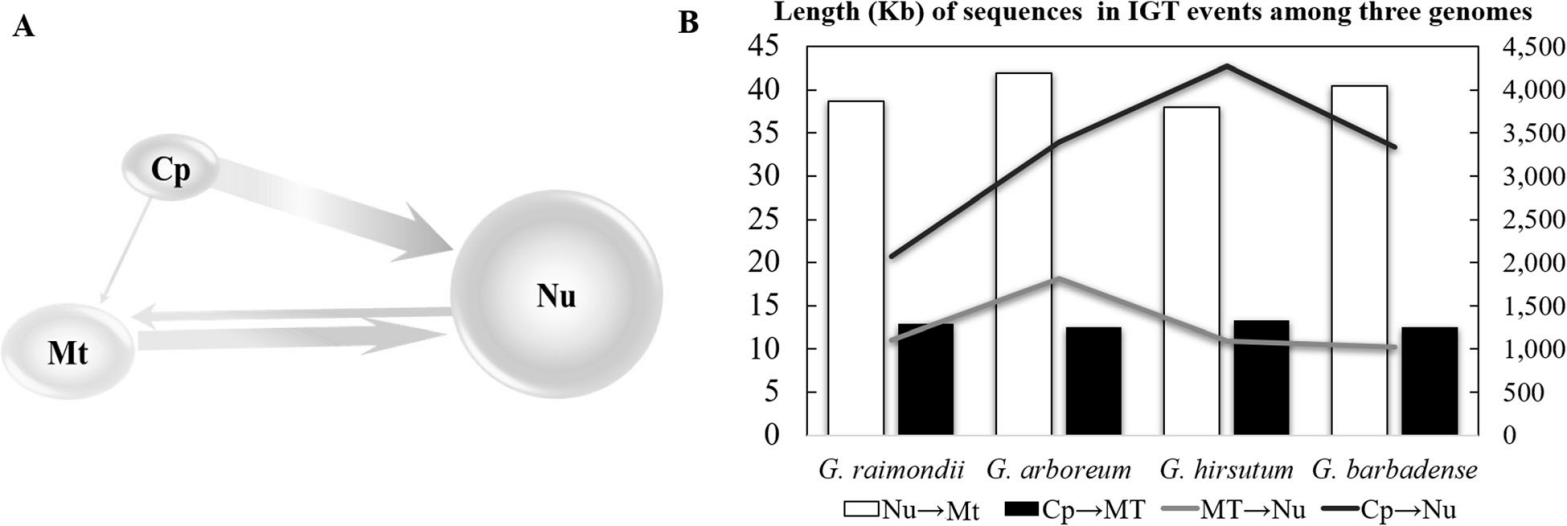
Intergenomic Gene Transfer (IGT) among the three genomes in four cotton species surveyed. **a** A model of four directional IGT events among three genomes where the arrow width represents the abundance of transfers in that direction. **b** Length of four directional IGTs among the three genomic compartments in four cotton species. Cp: chloroplast; Mt: mitochondrion; Nu: nucleus. The left scale bar represents the length (kb) of sequences transferring into mitochondrial genomes from nuclear (the lightest grey bars) and chloroplast genomes (the lighter grey bars); while the right scale bar denotes the length (kb) of sequences transferring into nuclear genomes from mitochondrial (the dark grey lines) and chloroplast genomes (the black lines). The horizontal axis lists four cotton species, *G. raimondii*, *G. arboreum*, *G. hirsutum,* and *G. barbadense*

We screened the nuclear, mitochondrial, and chloroplast genomes of four cotton species, two polyploids and their model diploid progenitors (Additional file 1), for evidence of IGT events. As expected, most of the detected IGTs involve four of the six possible classes of IGT events: nucleus-to-mitochondrion, chloroplast-to-mitochondrion, mitochondrion-to-nucleus, and chloroplast-to-nucleus (Fig. 1a). We did not detect nuclear or mitochondrial insertions into the chloroplast in any of four cotton species.

In the four cotton species surveyed, the length of the integrated fragments was typically 100 times longer for nuclear versus mitochondrial integrants. The size of nuclear integrants ranged from 1028 kb to 4276 kb, whereas mitochondrial integrants varied in size from only 13 kb to 42 kb (Fig. 1b, Table 1). Nuclear insertions into the mitochondrion were typically three times the length of chloroplast insertions (40 kb vs 13 kb; Table 1), and chloroplast insertions into the nucleus (*nupts*) were two to four times greater than mitochondrial insertions (*numts*). With the exception of *G. arboreum*, *numt* length was approximately equivalent among species (Fig. 1b), whereas *nupt* length varied about twofold (from 2072 kb in *G. raimondii* to 4276 kb in *G. hirsutum*; Table 1). Interestingly, the ratio of *nupt* to *numt* was 1.87 for both diploid cotton species and nearly double that (average 3.58) for the two allotetraploid cotton species (Table 1).

**Table 1:** The total length of IGT events among three genomes in four cotton species

Species	Mitochondrial integrants			Nuclear integrants		
	Nu→Mt (kb)	Cp → Mt (kb)	Nu→Mt/Cp → Mt	Mt → Nu (kb)	Cp → Nu (kb)	Cp → Nu/Mt → Nu
*G. raimondii*	38.74	12.89	3.01	1106.78	2072.12	1.87
*G. arboreum*	41.94	12.54	3.34	1813.38	3391.15	1.87
*G. hirsutum*	37.99	13.36	2.84	1092.08	4275.84	3.92
*G. barbadense*	40.50	12.54	3.23	1028.21	3331.78	3.24
Average	39.79	12.83	3.11	1260.11	3267.72	2.72

### Mitochondrial integrations are variable for nuclear repeats and conserved for chloroplast tRNA genes

**Fig. 2:**
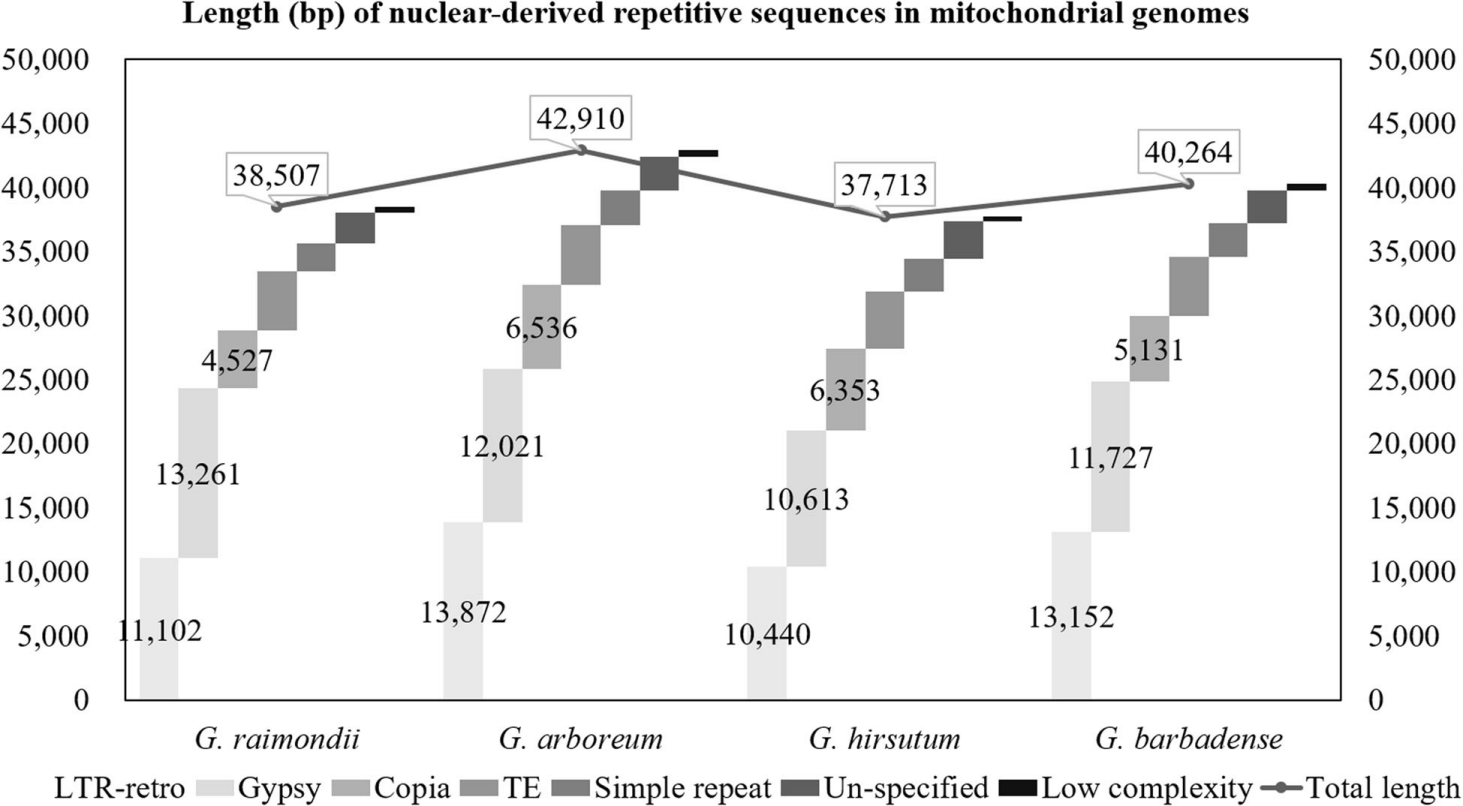
Length (bp) of nuclear-like transposable elements in mitochondrial genomes of four cotton species. The horizontal axis represents four cotton species. The vertical axis denotes the lengths of the nuclear-to-mitochondrial repeats including seven categories. The small rectangles in different shades of gray are symbols of seven kinds of repeats. The points in the line describe the total length of repeats in four cotton species. The bars and the lines refer to the left and the right coordinates, respectively. TE: transposable element that cannot be assigned to other detailed categories, mainly DNA transposable element; LTR-retro: long terminal repeats retrotransposons that cannot be assigned to either *gypsy* or *copia* classes

Repetitive sequences are common to the mitogenomes of various seed plants, including the monocot *Oryza sativa* [55] and the eudicots *Arabidopsis thaliana* [33], *Cucumis melo* [31], *Cucumis sativus* [28] and *Gossypium* species [60, 91]. Long terminal repeat (LTR) retrotransposons (LTR-retro) typically comprise the biggest component of plant nuclear repeats [94, 95], and they often are a predominant influence on nuclear and mitochondrial genome size [96–99]. Here, we identified nuclear-derived repeats in the mitochondrial genomes of all four cotton species. The total length of nuclear-derived repeats (for all repetitive classes) ranged from 37.7 kb in the *G. hirsutum* mitochondrial genome to over 42.9 kb in *G. arboreum* (Fig. 2), suggesting that nuclear-derived repeats comprise between 5.64 – 6.24% of a given cotton mitochondrial genome. These repeats were partitioned into seven classes: *copia*, *gypsy*, low complexity, unclassified long terminal repeat retrotransposon (LTR-retro), simple repeat, transposable element (TE) and unspecified (Fig. 2). Here, *copia* and *gypsy* represent a further partitioning of the general LTR-retro (Class 1) transposable elements into their two main classes [100]. The rank of each class (based on element abundance) was nearly identical for each species, with unclassified LTR-retro and *gypsy* elements contributing the most sequence in all four species (Fig. 2); however, the total sequence length for each class varied somewhat among species, leading to the approximately 5 kb difference in total nuclear-derived repeat length noted above. Neither the total amount of repetitive sequence nor the individual length of sequence per repeat class exhibited evidence of bias with respect to ploidy.

**Fig. 3:**
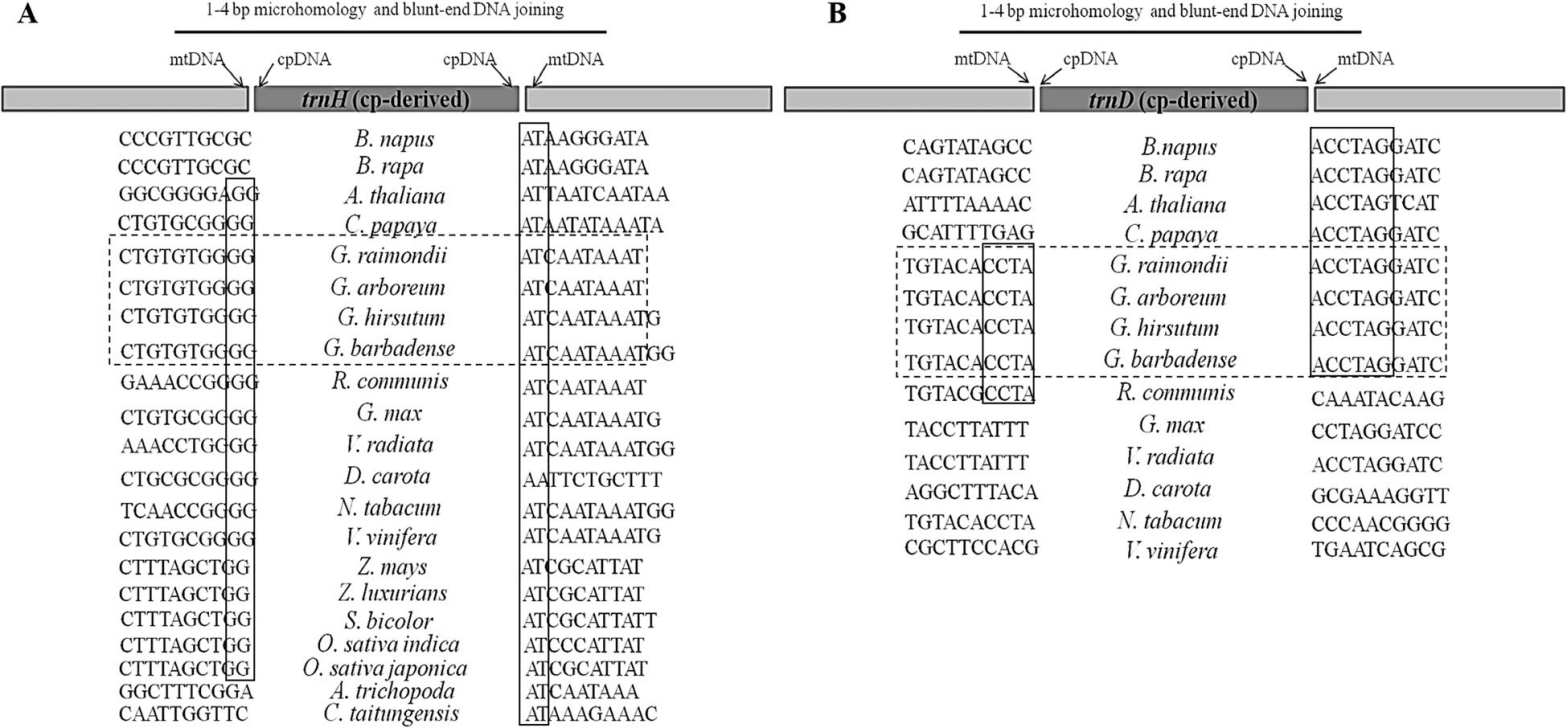
Flanking nucleotide analysis of chloroplast-derived genes, *trnH* (**a**) and *trnD* (**b**), in mitogenomes. cpDNA and mtDNA are the abbreviations of chloroplast DNA and mitochondrial DNA. The light-dark-light gray strip represents the fusion sequence of mtDNA-cpDNA-mtDNA. The capital English letters in the long rectangular boxes, such as “GG” and “AT”, close to cpDNA show the nucleotides of micro-homologies among the different species. Dotted rectangles enclose flanking sequences for the four cotton species studied here

In addition to incorporating nuclear repeats, the presence of chloroplast-like tRNA genes in plant mitogenomes has been described [27, 34, 65]. In our previous research, we identified eight chloroplast-derived tRNA genes (*trnD*, *trnH*, *trnM*, *trnN*, *trnP*, *trnS*, *trnV* and *trnW*) in the mitogenomes of all four cotton species [65]. The presence of these genes in species dispersed on the cotton phylogeny likely indicates that their transfer occurred in a common ancestor and that they have been preserved. To evaluate this suggested history, we analyzed nucleotides flanking insertion sites using two chloroplast-derived tRNA genes (*trnH* and *trnD*) as examples. For both genes, we found that the 10 bp upstream and downstream of the insertion were shared among the four cotton species (Fig. 3). When we broadened the number of plant species to include diverse angiosperms, we discovered shared 2-6 bp microhomologies flanking the insertion sites of these two genes (Fig. 3).

Additionally, we evaluated the contribution of both nuclear and chloroplast derived sequences to overall mitochondrial genome size for 26 land plants. Although both nuclear and chloroplast sequences have contributed to mitogenome expansion, the total length of nuclear-like sequences in mitogenomes is more strongly correlated with mitogenome size variation (*R*^2^ = 0.77) than is the length of chloroplast sequences (*R*^2^ = 0.36) (Additional file 2A, B). This is partly due to variation in the total amount of repetitive sequence inserted into the mitogenome; however, this correlation is weak (*R*^2^ = 0.13) (Additional file 2C), as is the correlation between total repeat length and total nuclear/chloroplast length (*R*^2^ = 0.23 and 0.0048; Additional file 2D, E).

### Nuclear insertion of mitochondrial DNAs (*numts*) and chloroplast DNAs (*nupts*)

**Fig. 4:**
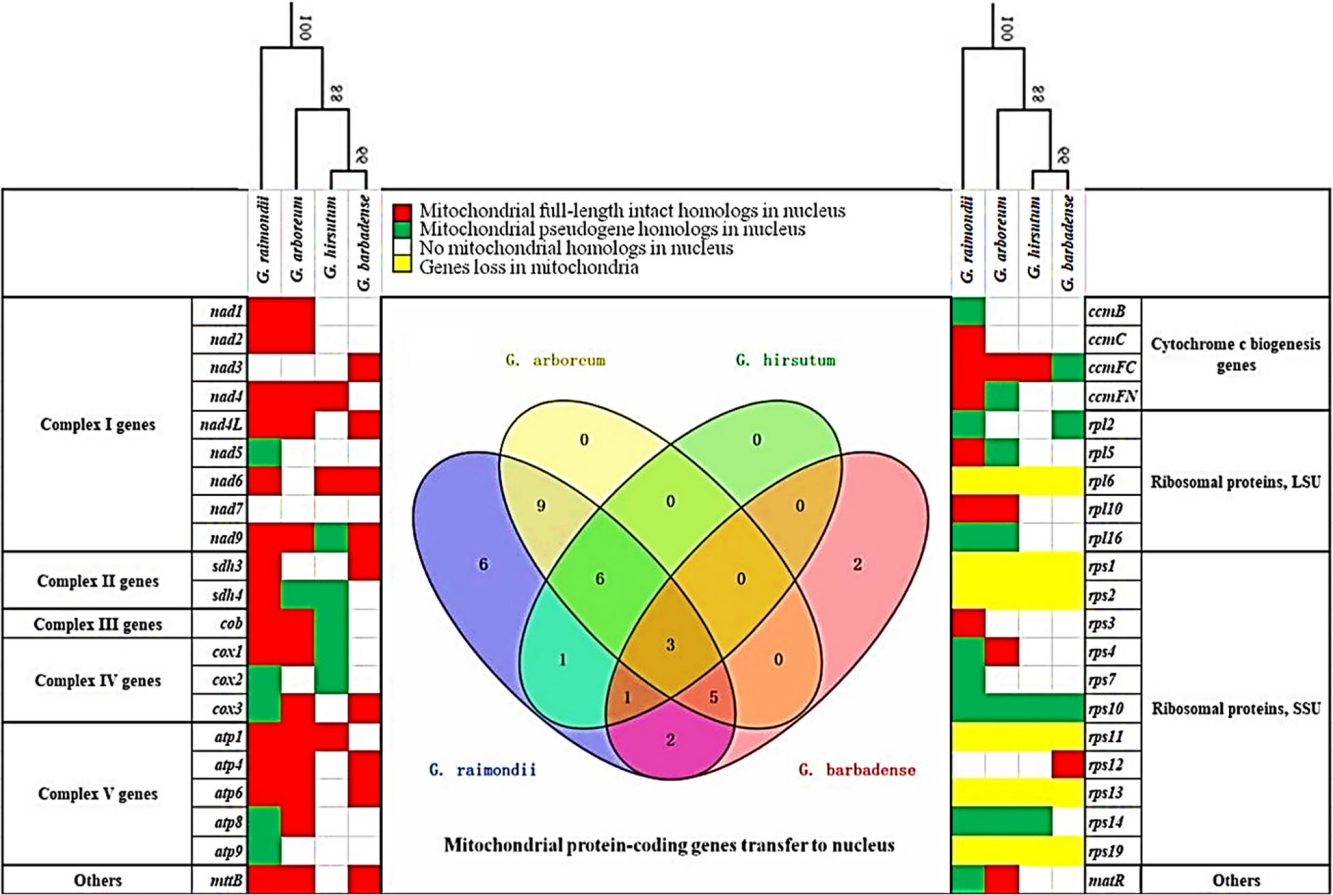
Mitochondrial gene transfers and losses among four cotton species. The first set columns on the left and the set on the right are mitochondrial protein-encoding genes, their functional categories, and their presence/absence in the nucleus and/or mitochondrion. The first line lists the names of plant species, with their relationships designated by the tree. The colored boxes represent the presence of: (1) mitochondrial full-length intact homologs in the nucleus (red); (2) mitochondrial pseudogene in the nucleus (green); (3) absence of mitochondrial homolog in the nucleus (white); and (4) complete absence of that gene in the mitochondrion (yellow). Center: Venn diagram of the mitochondrial protein-coding genes transferred into the nuclear genome (corresponding to the red and green cells on the left) from *G. arboreum* (yellow), *G. raimondii* (blue), *G. hirsutum* (green)*,* and *G. barbadense* (red), respectively. The overlap among circles shows the common *numts* among those species

We evaluated the presence-absence pattern of 42 common mitochondrial protein-coding genes for the four cotton species studied, in both the mitochondrion and nucleus, to provide insight into nuclear-mitochondrial coevolution. As expected, most mitochondrial genes were still present in mitogenomes. Only six ribosomal subunit genes (*rpl6*, *rps1*, *rps2*, *rps11*, *rps13* and *rps19*) were absent in all four cotton species (Fig. 4, yellow cells), which likely represent shared loss at some point in their evolutionary history. For mitochondrial genes encoding both complex II (succinate dehydrogenase, *sdh* genes) and ribosomal subunits (*rpl* and *rps* genes) *Gossypium* retains more genes (e.g., *sdh3* and *sdh4*, *rpl10* and *rps10*) in its mitochondrial genome. All remaining mitochondrial genes (except *nad7*) experienced full- or partial-length transfer to the nucleus in at least one cotton species, (Fig. 4, white cells).

Within *Gossypium*, the number of *numts* in diploids is two to three times that found in tetraploids (33 in *Gossypium raimondii* and 23 in *G. arboreum*, versus 11 in *Gossypium hirsutum* and 13 in *G. barbadense*; Fig. 4). Many of the diploid *numts* (23) are shared between the two diploids, *G. raimondii* (D_5_) and *G. arboreum* (A_2_), which indicates that these *numts* were incorporated into the nuclear genome prior to the divergence of the A and D clades at the base of *Gossypium.* Subsequently, the lineage leading to *G. raimondii* acquired a further 10 *numts* (*nad5*, *atp9*, *ccmB*, *ccmC*, *rps3*, *rps7*, *nad6*, *cox2*, *sdh3* and *rpl2*). In contrast, the two tetraploids (*G. hirsutum* and *G. barbadense*) suffered massive, differential *numt* decay after allotetraploidization. Only three of the shared diploid *numts* (*nad9*, *ccmFC,* and *rps10*) and one D_5_-specific *numt* (*nad6*) are present in both *G. hirsutum* and *G. barbadense*. *Gossypium hirsutum* contains an additional six shared diploid *numt* genes (*nad4*, *sdh4*, *cob*, *cox1*, *atp1* and *rps14*) and one D_5_-specific *numt* gene (*cox2*). On the other hand, *G. barbadense* contains five different diploid-common*numts* (*nad4L*, *cox3*, *atp4*, *atp6* and *mttB*), two different D_5_-specific *numts* (*sdh3* and *rpl2*), and two AD_2_-specific *numts* (*nad3* and *rps12*).

Interestingly, most *numts* remain intact (full length) after insertion (Fig. 4, red cells), with few that degraded to truncated pseudogenes (Fig. 4, green cells). In a few cases (i.e., *cox2*, *rpl2*, *rpl16*, *rps10* and *rps14*), there is evidence of pseudogenes in diploids and polyploids. The remaining 10 pseudogenes (*nad9*, *sdh4*, *cob*, *cox1*, *cox3*, *atp8*, *ccmFC*, *rpl5*, *rps4,* and *matR*) were intact in diploids but truncated in polyploids. Additionally, four *numt* pseudogenes (*nad5*, *atp9*, *ccmB,* and *rps7*) occurred only in *G. raimondii* (Fig. 4, green cells).

**Fig. 5:**
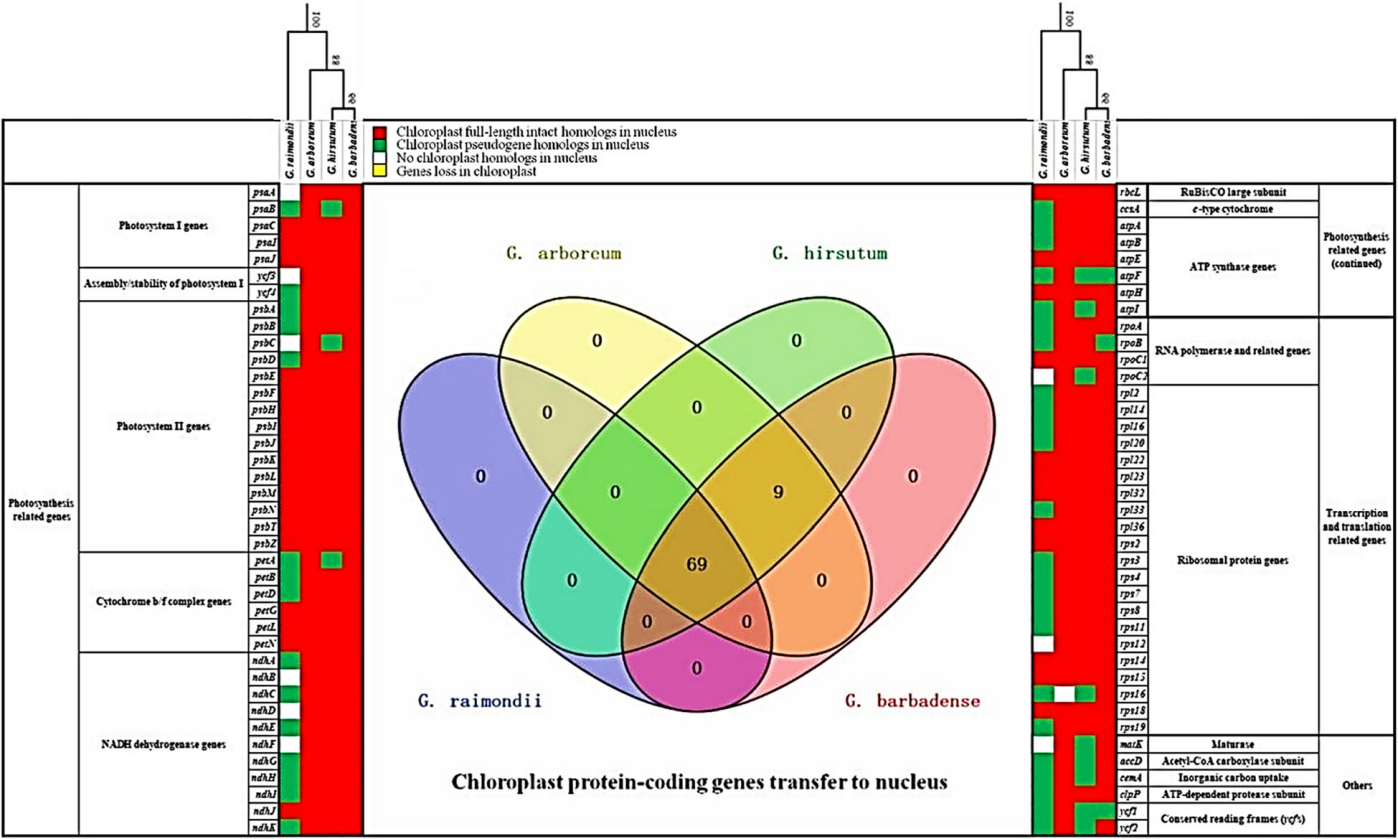
Chloroplast genes transfers to the nuclear genome in four cotton species. The columns on the left and right represent the functional categories and names of chloroplast-encoded genes. The first line lists the names of plant species and the phylogenetic relationship above. The red and green cells represent chloroplast full-length intact homologs and pseudogenes in nuclear genomes, respectively. White and yellow cells represent no chloroplast homologs in nuclear genomes and genes lost from chloroplast genomes, respectively. Center: Venn diagram containing all chloroplast protein-coding genes transferred into the nuclear genome (corresponding to the red and green cells) in *G. arboreum* (yellow), *G. raimondii* (blue), *G. hirsutum* (green)*,* and *G. barbadense* (red). The overlap among circles shows the common *nupts* among those species

We evaluated each genome for nuclear insertions of all 78 chloroplast genes (*nupts*) in the four cotton species. None of the 78 chloroplast genes were lost from the plastids (Fig. 5, yellow cells), and almost all of the 78 genes experienced transfer to nucleus in at least one of the four cotton species. Only six *nupts* (*psaA*, *ycf3*, *psbC*, *ndhB*, *ndhD*, *ndhF*, *rpoC2*, *rps12* and *matK*) in *G. raimondii* (D_5_) and one *nupt* (*rps16*) in *G. arboreum* (A_2_) were absent (Fig. 5, white cells).

In contrast to the greater abundance of *numts* in *G. raimondii*, *nupts* experienced a general decay in *G. raimondii* relative to the remaining cotton species (Fig. 5)*.* Nearly all of the 78 *nupts* are present in *G. arboreum*, *G. hirsutum*, and *G. barbadense* (except for *rps16 nupt* loss in *G. arboreum*) as full or partial *nupts*; 69 *nupts* are common to the four cotton species (Fig. 5). In general, *G. arboreum* (A_2_) retains the most *nupts* (77 out of 78), whereas there was considerable *nupt* degradation (39) or removal (9) from the *G. raimondii* genome. As in *G. arboreum, nupts* in *G. barbadense* are typically retained as intact (75/78) whereas in the other polyploid species, *G. hirsutum,* there has been a modest amount of degradation (12/78 are degraded; Fig. 5). One *nupt* (*rps16*) that is absent in *G. arboreum* is a pseudogene in *G. raimondii* and *G. hirsutum*, only presenting as intact in *G. barbadense*. This suggests that this *rps16 nupt* was transferred to the nucleus sometime during the evolution of *G. raimondii* after divergence from the *G. arboreum* lineage, and that it was subsequently retained in polyploid *Gossypium* until it experienced decay in the lineage leading to *G. hirsutum*. In *G. barbadense*, the three *nupts* (out of 78) that have experienced degradation (i.e., *atpF*, *rpoB,* and *ycf1*) all experienced IGT in the common ancestor of the diploid species (*G. raimondii* and *G. arboreum*), and experienced differential degradation in *G. raimondii* and both allopolyploids (except the intact *rpoB* in *G. hirsutum*). Degradation of *nupts* was more prominent in *G. hirsutum*, where 15% of *nupts* were degraded (12/78; *psaB*, *psbC*, *petA*, *atpF*, *atpI*, *rpoC2*, *rps16*, *matK*, *accD*, *accmA*, *ycf1* and *ycf2*). Almost all of these (nine of 12) are also pseudogenes in *G. raimondii*, while chloroplast *psbC*, *rpoC2* and *matK* were absent in the nucleus. Overall, *G. raimondii* experienced the most degradation of *nupts*, where over half (39 out of 78) were decayed and nine were not present (i.e., *psaA*, *ycf3*, *psbC*, *ndhB*, *ndhD*, *ndhF*, *rpoC2*, *rps12* and *matK*).

**Fig. 6:**
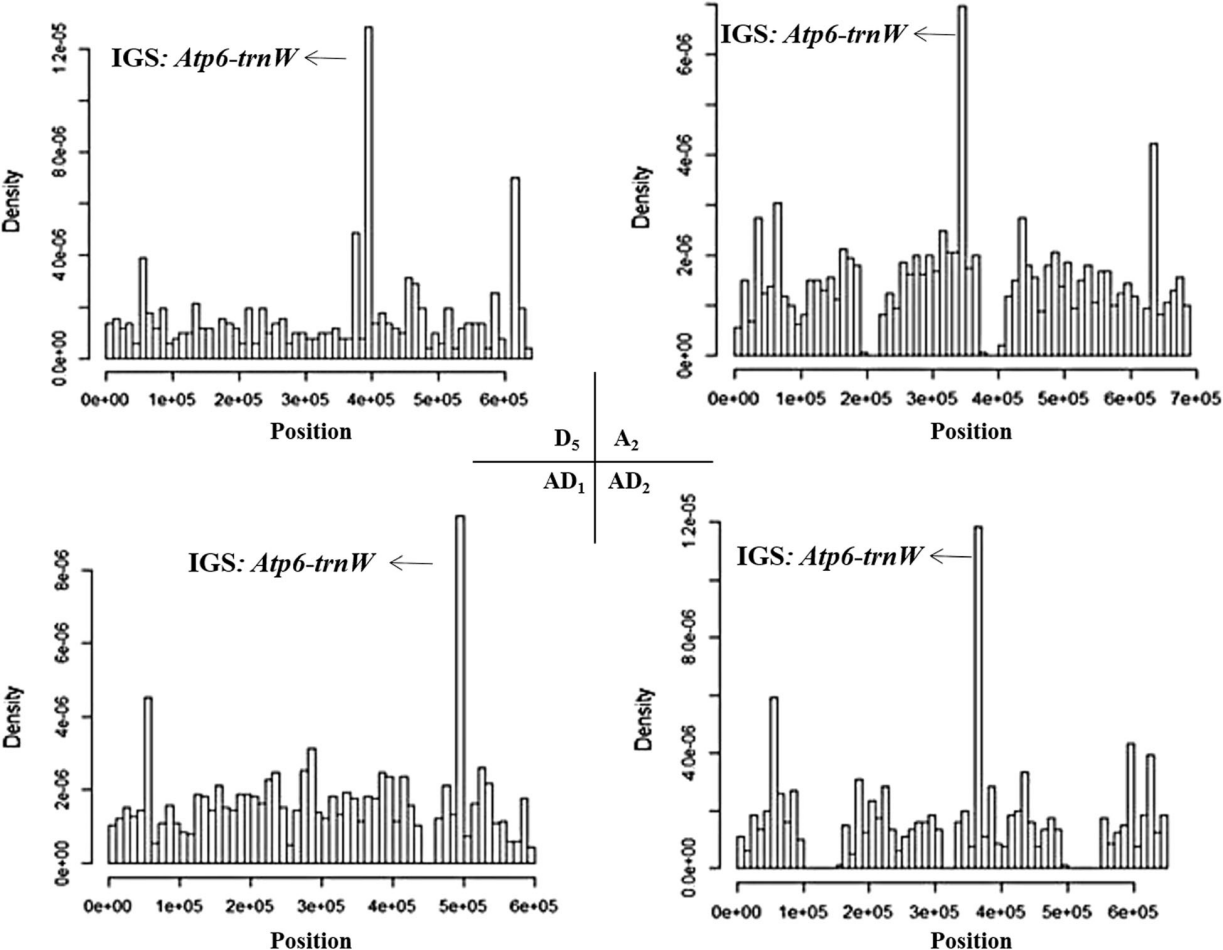
Mitochondrion-to-nucleus transfer-out hotspots in four cotton species*.* IGS: intergenic sequence. The top left corner shows the transfer hotspot of mitochondrial genomes in *G. raimondii* (D_5_). The top right corner shows the transfer hotspot of the mitochondrial genomes in *G. arboreum* (A_2_). The bottom left corner shows the transfer hotspot of mitochondrial genomes in *G. hirsutum* (AD_1_). The bottom left corner shows the transfer hotspot of mitochondrial genomes in *G. barbadense* (AD_2_). The horizontal axis indicates the nucleotide position in each mitochondrial genome. The density of mitochondrial-to-nuclear transfers is along the vertical axis. The arrows show the maximum density of IGT frequency

**Fig. 7:**
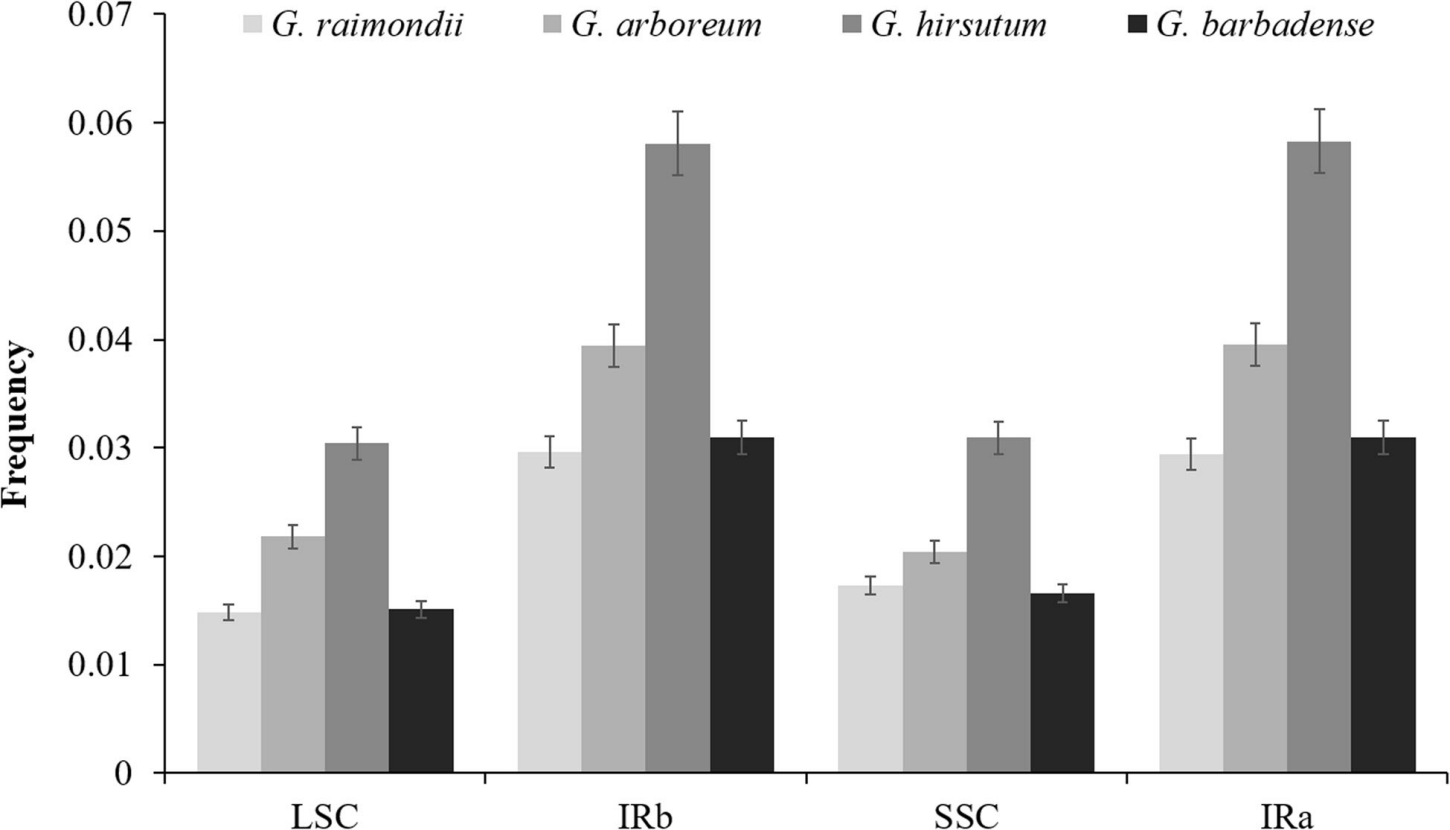
Chloroplast-to-nuclear transfer hotspots in chloroplast genomes of four cotton species*.* LSC: large single copy region. IR: inverted repeat region. SSC: Small single copy region. Four cotton species are shown in different shades of gray. The frequency of chloroplast-to-nuclear transfers is along the vertical axis. The bars represent the frequency of sequences transferring from the chloroplast to the nucleus of four cotton species. The error bars represent +/− 5%

Interestingly, not all regions of the chloroplast and mitochondrial genomes transferred with equal frequency. A hotspot of mitochondrial source material was located in the *atp6*-*trnW* intergenic region (Fig. 6), and in the chloroplast genome there were three hotspots, i.e., the large single copy region (LSC), inverted repeat region (IR), and small single copy region (SSC), these transferred at a relative rate of 1: 2: 1 (Fig. 7).

Congruent with most angiosperm species, the majority of norgDNAs in cotton are small to medium in size (100 bp – 5 kb) (Additional files 3 and 4), and their distribution patterns vary among species (Additional file 3). Two previously noted complete mitochondrial genome transfers were found on Chr01 of *G. raimondii* and ChrA03 of *G. hirsutum* [64, 93], similar to large-scale norgDNAs found in other plants, e.g., the *numt* on Chr2 of *A. thaliana* [61, 101] and the *nupt* inserted into one scaffold of *S. bicolor* (Additional file 3)*.* The number of reverse matches (the direction of the sequence intervals along the genome coordinates in donor and receptor genomes are reverse, the start-to-end position of one genome is from small number to large number, while the other genome from large to small) is no fewer than that of the positive matches (both positions in donor and receptor genomes are from small to large, or from large to small). Two kinds of matches coexist in the same chromosome in most studied species. The *nupts* of six chromosomes (chr01, chr03, chr05, chr06, chr08 and chr09) are all reverse matches (blue dots or fragments) and the other seven chromosomes positive matches (red dots or fragments) in *G. raimondii*. Third, norgDNAs in some species frequently transfer into certain chromosomes, but such nuclear hotspots of integration vary from species to species, like *numts* on Chr2 of *A. thaliana,* Chr01 of *G. raimondii,* and ChrA03 of *G. hirsutum*. Fourth, the transfer of norgDNAs is phylogenetically sporadic, as there often is little *norg* similarity between closely related species.

We examined the relationship between IGT into the nucleus and genome size, comparing this to the effects of repetitive content on genome size variation. While nuclear repeat size is most strongly correlated with genome size (*R*^2^ = 0.9813; Additional file 5C and Table 2), norgDNA length was moderately correlated with nuclear genome size (*R*^2^ = 0.5917 and *R*^2^ = 0.4675 for *numts* and *nupts*, respectively; Additional file 5A and B). Additionally, the length of nuclear repeats and norgDNAs was also moderately correlated (*R*^2^ = 0.6321 for *numts* and *R*^2^ = 0.4511 for *nupts*; Additional file 5D and E).

**Table 2:** Repeats variation of nuclear genomes in four cotton species

Species	Genome sizes (Mb)	Repeat sizes (Mb)	Repeat percentage (%)	*Numt* length (kb)	*Numt* percentage (%)	*Nupt* length (kb)	*Nupt* percentage (%)	References
*G. raimondii*	761.57	423.43	55.60	1107	0.15	2072	0.27	[64]
*G. arboreum*	1694.60	1160.80	68.50	1813	0.11	3391	0.20	[83]
*G. hirsutum*	2173.00	1460.26	67.20	1092	0.05	4276	0.20	[84]
*G. barbadense*	2570.00	1776.13	69.11	1028	0.04	3332	0.13	[88]

### Low expression levels of organellar genes in nucleus (norgDNAs)

**Fig. 8:**
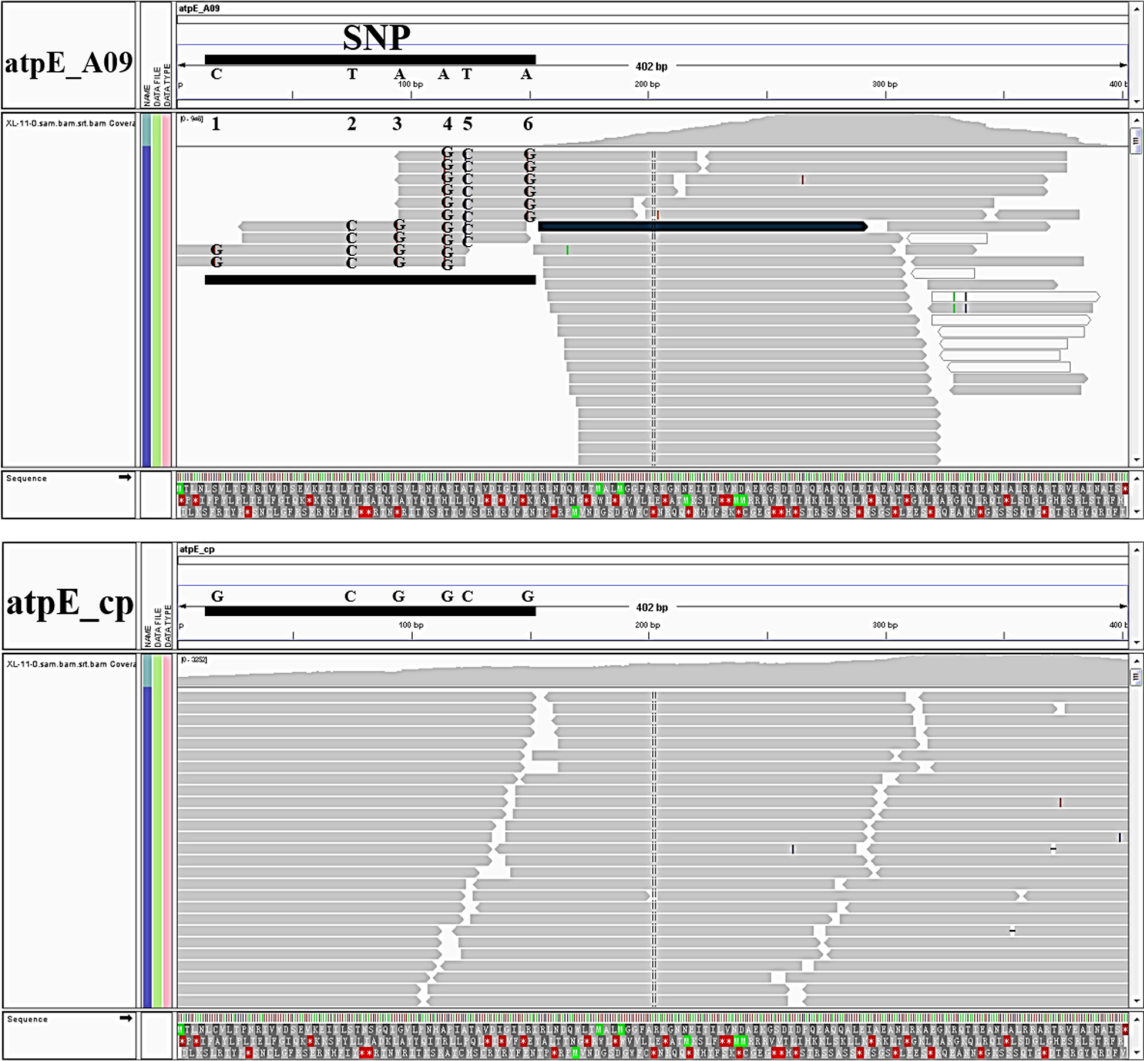
RNA-seq expressed paired-end reads of chloroplast gene *atpE*_cp and its nuclear homology *atpE*_A09 in one *G. hirsutum* variety (Xinluzao 11). The two images above show the variability and coverage of the RNA-seq expressed paired-end reads via an Integrative Genomics Viewer (IGV) screenshot. Each image contains three main panels. The upper panel represents the sequence coordinates. The middle panel is subdivided into two tracks, where the upper track depicts read density and the lower track represents the mapped clean reads. The panel at the bottom represents the linear DNA sequence. The SNPs in *atpE*_A09 and the corresponding normal nucleotide acids in *atpE*_cp are highlighted in bold. The expressed reads of *atpE*_A09 share the same nucleotide acid sequences with *atpE*_cp and few expressed reads are mapped to the region of divergence

To assess if the norgs in cotton are expressed, we analyzed leaf RNA-seq data from two accessions of *G. hirsutum,* i.e., X11 and X42. We found that the relative expression of organellar genes was generally much higher than their intact nuclear counterparts (Table 3), although one norgDNA (the *nupt atpE_*cp) had a relatively high RPKM value compared to other norgDNAs. To explore this further, we evaluated the amount and pattern of sequence variation differentiating the organellar gene from the norgDNA and the resulting expression difference. Only six SNPs differentiate the 402 nucleotides of *atpE*_A09 and *atpE*_cp (~ 1.5% sequence divergence), indicating that this is a relatively recent or highly conserved norgDNA. When we removed all reads that could not be accurately assigned to *atpE*_A09 or *atpE*_cp, we found that no reads actually mapped to the region distinguishing the nuclear and organellar copies of *atpE* (Fig. 8). When we repeat this partitioning for an additional chloroplast gene (*petG*_cp) whose percent divergence between nuclear and chloroplast copies was sufficiently high to distinguish the two copies (~ 6.1% divergence, fourfold greater than *atpE*_A09), relatively few reads were both ambiguously mapped and assigned to a nuclear origin (Additional file 6). While this suggests that our RNA-seq analyses are suitable for capturing the expression differences between organellar and norg genes, we also validated these interpretations via qRT-PCR (Additional file 7) using *atpE* and *petG* as exemplars.

**Table 3:** Transcription levels of 14 organellar genes and nuclear homologs in two *G. hirsutum* varieties

Organellar genes/ Nuclear homologies	DNA lengths (bp)		RPKM value_X42^a^		RPKM value_X11^a^		SNP numbers
	OrgDNA	NuDNA	OrgDNA	NuDNA	OrgDNA	NuDNA	
*atpE*_cp*/atpE*_A09	402	402	661.56	71.19	1219.42	165.73	6
*ndhC*_cp*/ndhC*_D07	363	363	90.29	2.95	177.01	3.45	12
*ndhE*_cp*/ndhE*_A01	306	306	27.38	0.00	52.07	0.65	22
*ndhK*_cp*/ndhK*_A10	678	678	156.19	1.68	257.03	3.50	21
*petG*_cp*/petG*_D12	114	114	129.93	1.18	199.81	2.31	7
*petL*_cp/*petL*_D01	96	96	154.29	7.68	378.55	17.14	6
*petN*_cp*/petN*_D04	90	90	75.96	0.00	112.65	0.00	5
*psaJ*_cp*/psaJ*_D02	129	129	560.61	3.64	1214.12	5.61	6
*psbE*_cp*/psbE*_D06	252	252	184.58	0.53	230.42	0.26	14
*psbH*_cp*/psbH*_A05	222	222	63.70	8.15	105.87	7.41	9
*sdh4*-mt*/sdh4*_D04	399	399	8.90	1.85	9.07	5.11	1
*rps14*-mt*/rps14*_A01	303	303	25.44	0.00	21.29	0.22	8
*nad6*-mt*/nad6*_A08/A03	621	621/621	8.42	0.00/3.78	7.00	0.00/2.54	33/1
*cob*-mt*/cob*_A01	1179	1179	7.50	0.11	7.15	0.00	27

^a^RPKM values were calculated by RNA-seq data; X11 and X42 represented two *G. hirsutum* varieties, Xinluzao 11 and Xinluzhong 42

### NorgDNAs changes during the process of diploids and allopolyploids evolution

**Fig. 9:**
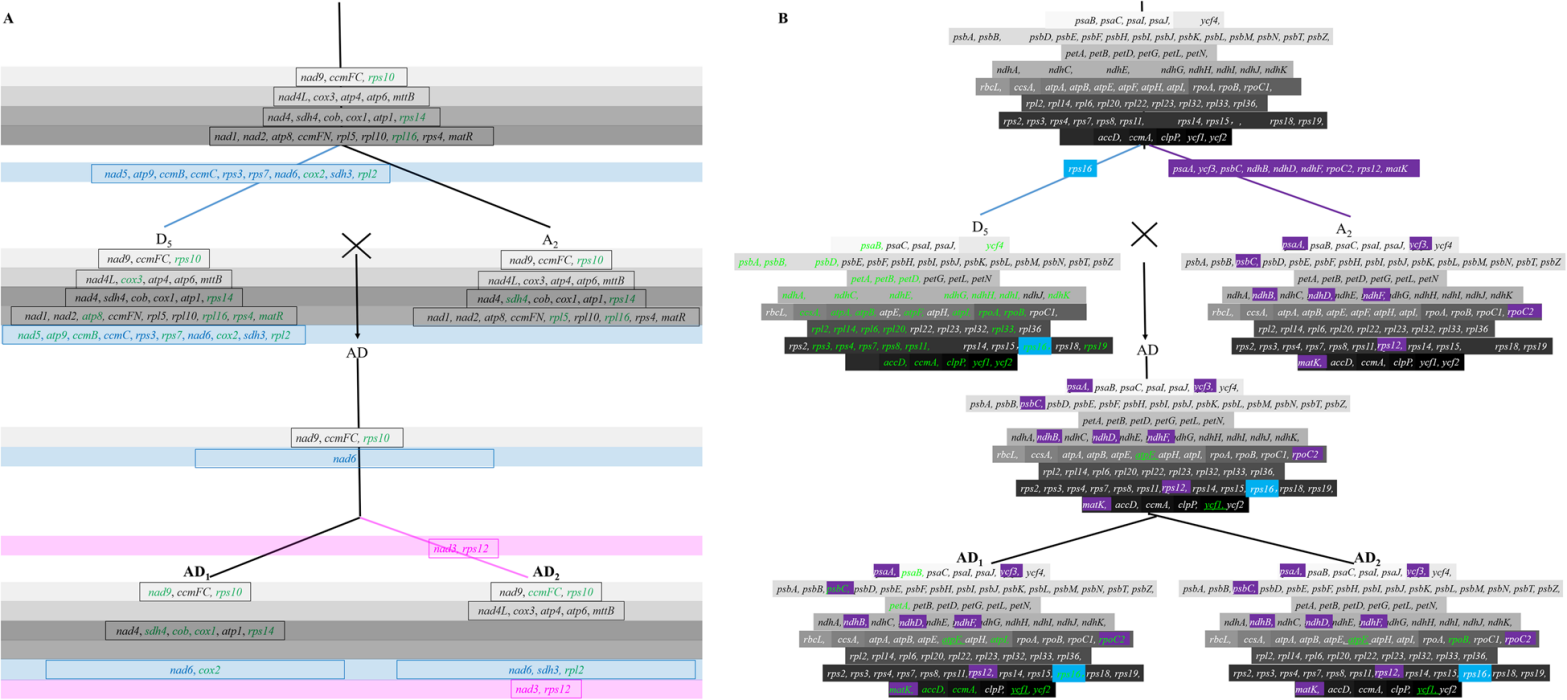
Hypothetic evolutionary model showing the change of norgDNAs during allopolyploidization of cotton species. **a** Schema graph showing the mitochondrial-to-nuclear IGT events during the allopolyploidy of *Gossypium*. Genes in green color denote pseudogenes. Genes in blue rectangular strip transferred only in D_5_ after the differentiation of D_5_ and A_2_. Genes in purple rectangular strip transferred only in AD_2_ after the differentiation of AD_1_ and AD_2_. **b** Schema graph showing the chloroplast-to-nuclear IGT events during the allopolyploidy of *Gossypium*. Genes in the same gray boxes belong to one functional classification. Genes in green color denote pseudogenes. The genes in blue and purple boxes transferred after the differentiation of D_5_ and A_2_ genomes

>For IGT into the nucleus, the impact of allopolyploidy is notable and appears different for mitochondrial-to-nuclear IGT and chloroplast-to-nuclear IGT. For *numts*, fewer transfers are inferred in the allopolyploid species relative to their diploid progenitors, and the *numts* present are more frequently partial and/or decayed copies (Fig. 9a). Conversely, almost all *nupts* present in the diploids are retained in the allopolyploid species (Fig. 9b). To evaluate if this pattern is repeated for other polyploid systems, thereby suggesting that this might be a general phenomenon associated with genome doubling, we analyzed IGT for allopolyploid *Brassica napus* (AACC) relative to its model diploid progenitors, *B. oleracea* (CC). *B. rapa* (AA). Unlike *Gossypium*, allopolyploid *Brassica* shows similar rates of integration for both *numts* and *nupts* (Additional file 8).

## Discussion

### Variable rates of IGT occur between the intracellular genomes of *Gossypium*

In our study, we characterized the rate and direction (i.e., nucleus ↔ chloroplast, nucleus ↔ mitochondrion, chloroplast ↔ mitochondrion) of IGT between the intracellular genomes of four *Gossypium* species. We detected most mitochondrial genes in present mitogenomes, which suggest that most genes (i.e., protein-encoding, rRNA, and tRNA genes) in *Gossypium* mitochondrial genomes are much conserved [29, 102–104]. For example, the mitochondrial genome of *Gossypium* retains some genes encoding both complex II (succinate dehydrogenase, *sdh* genes) and ribosomal subunits (*rpl* and *rps* genes) that have been massively lost in other angiosperms [9, 17], e.g., *sdh3* and *sdh4*, *rpl10* and *rps10*. Meanwhile, we found all chloroplast genes in any of the four cotton species’ plastids, which validates the general conservation of chloroplast genes [105], as in most plant species [9]. While all three intracellular genomes were involved in IGT, the only directions of IGT not detected were nuclear or mitochondrial transfers into the chloroplast. This is consistent with observations from most other plants [9], with two notable exceptions, i.e., *Daucus carota* [39] and *Asclepias syriaca* [40], where intracellular transfers into the chloroplast have been reported. Together, these observations suggest that the plastid genome lacks an active mechanism to integrate exogenous sequences.

Overall, we found substantially more sequences were integrated into the nucleus than the mitochondrion, a phenomenon that may be due to the mechanisms by which sequences get integrated into each genome, limitations on mitochondrial genome size, or both. Nearly all mitochondrial and chloroplast genes experienced transfer to the nucleus in *Gossypium*, which is a pattern seen in most other angiosperm plants [9], and at a transfer rate that is 100-fold greater than into the mitochondrial genome.

### Non-additive effects of allopolyploidization on IGT in *Gossypium*

Genome doubling via polyploidy has numerous consequences [106]. Polyploidy can alter the size, content, and complexity of the genome [107], consequently affecting genetic variation, stress adaptation, biological complexity, speciation, biodiversity [108] and evolutionary novelty [109]. Myriad genomic consequences have been documented for polyploidy [110–112]; however, the cyto-nuclear effects of allopolyploidy in particular (which results from hybridization of divergence species) have been underexplored [113] and little is known regarding the influence of polyploidy on IGT. Here, we evaluate IGT transfers for two allopolyploid species, *G. hirsutum* and *G. barbadense,* which arose from a single polyploidization event about 1–2 million years ago involving the ancestors of the two diploid cottons sequenced here [81]. We found one diploid ancestral species (here, *G. raimondii*) experiences more *nupt* truncation and/or loss than the other diploid ancestral species (here, *G. arboreum*), and the resulting polyploid retains nearly all of the *nupts* found in the diploids, similar to the extensive retention found in *G. arboreum*. These patterns are consistent with the general observation found from a survey of 21 land plants [9]. Conversely, mitochondrial-to-nuclear IGT are massively lost in the allopolyploid species. That is, only a few decayed *numts* are retained in the allopolyploid species, less than retained in either diploid progenitor, and those that were retained were more commonly the older *numts* shared between diploid species.

The total length of retained *numts* and *nupts* did not approach additivity for either polyploid species, *G. hirsutum* (AD_1_) or *G. barbadense* (AD_2_), with the possible exception of *nupts* in *G. hirsutum* whose total length was 80% of that found in their representative model diploid parents, *G. raimondii* (D_5_) and *G. arboreum* (A_2_). This may reflect insertions in the model diploid parents, either after divergence from the polyploid or the true polyploid progenitors, or it may represent differential decay in the polyploids, both upon formation and over time. Interestingly, the ratio of *nupt* to *numt* in the two allotetraploid cotton species was twice that in both diploid cotton species, indicating a possible shift in relative *nupt* and *numt* incorporation and/or retention (toward *nupt*) in polyploid cotton. This could be partially explained by the challenge in uniquely identifying mitochondrial-derived repeats from the background repeats of the nuclear genome, particularly as these degrade over time. Our preliminary results (above) suggest similar norgDNA integration rates in *Gossypium* and *Brassica*, but more data are needed to understand the influences of ploidy on *nupt* and *numt* integration and degradation dynamics. While this hints at the differences among allopolyploid systems with respect to IGT, it is premature to draw general, more widely applicable conclusions. Clearly, this area requires further study to understand the evolutionary implications of norgDNAs, the patterns and processes by which they evolve in different biological systems, and the influences of ploidy on integration and degradation.

### Patterns of IGT among intracellular genomes

Here we found that the mitochondrial *atp6*-*trnW* intergenic region and the chloroplast inverted repeat region represent hotspots for IGT source material, i.e., these regions were frequently transferred to other intracellular genomes. In the mitochondrial genome, these (and other) transfers were frequently associated with 10 bp microhomologies, a phenomenon that was observed across distantly related genera (as 2-6 bp microhomologies).

Detected *numts* were found in various states of completeness, either due to the length during transfer or subsequent decay. Here we found that most *numts* remain full-length after insertion, suggesting that the mechanism responsible for *numt* generation may preferentially operate on full length genes; however, a few genes did transfer to the nucleus as partial sequences, indicating that partial genes are not excluded from transfer. Using a phylogenetic approach, we also detected genes that were transferred intact but decayed afterwards, e.g. *nad9* and *atp8*. Because younger *numts* are more readily identified and detection becomes more difficult as the *numt* decays, there is a natural bias toward detection of younger, more intact, and/or conserved numts, decaying *numts/nupts* slowly becoming increasingly difficult to detect as they lose sequence similarity to their source. Here, however, we describe several *numts/nupts* that have survived the basal-most radiation of the genus, approximately 5-10 MYA. Further insight into the potential reasons for these uncommon retentions will require additional functional study.

### Contributions of IGT to genome expansion

The underlying causes of genome size variation represent an old question for the nuclear genome and a relatively recent one for the mitochondrial genome. With respect to the latter, we found that both nuclear and chloroplast sequences are correlated with mitogenome expansion, concordant with the view that contamination of plant mitochondrial with nuclear and chloroplast DNA is at the heart of mitogenome expansion in plants [114]. As most plant genomes are composed of massive amounts of repetitive sequence, it is tempting to suggest that nuclear-derived repeats should represent the most frequent transfers; however, correlations between repetitive sequence characteristics (e.g., number, total amount) and the mitogenome size were weak. Therefore, although nuclear repeat-derived transfers do contribute to mitogenome size increase, they cannot fully explain the correlation between nuclear-to-mitochondrial transfer and mitogenome expansion.

With respect to nuclear genome size variation, it is commonly accepted that repetitive content underlies most of the size variations among species; however, the contribution of other sources of genome size expansion are less well characterized. Here we found that while nuclear repeats do contribute the most (more than half) to genomes size differences among species (i.e., 55.60, 68.50, 67.20 and 69.11% in *G. raimondii*, *G. arboreum*,*G. hirsutum* and *G. barbadense*, respectively), the contribution of *numts* and *nupts* to genome size is not insignificant (Table 2). Presence of *numts* and *nupts* were both positively correlated with nuclear genome size, with *nupts* affecting the genome size to a somewhat greater extent (i.e., 0.27, 0.20, 0.20 and 0.13% in *G. raimondii*, *G. arboreum*, *G. hirsutum* and *G. barbadense*, respectively) than *numt* doing (i.e., 0.15, 0.11, 0.05 and 0.04% in *G. raimondii*, *G. arboreum*, *G. hirsutum* and *G. barbadense*, respectively, Table 2)*.* We also found a positive correlation between nuclear repeats and norgDNAs, which may reflect a greater ability for norgDNAs to successfully integrate into genomes with larger gene-free regions.

### The consequences of organelle-to-nuclear transfers are largely unknown

Organelles have a history of functional transfers to the nucleus, some of which are conserved among distantly related lineages and others which are lineage specific. These are in addition to non-functional transfers, which may represent sequences varying in size and content from gene fragments to large regions of the organellar genome [4, 14, 18, 19, 46, 101, 115, 116]. While many recent organellar-derived sequences are inactive and/or nonfunctional, some transfers to the nucleus may have function [11, 16, 17, 117], and both functional and non-functional transfers can have consequences for intracellular metabolism and genome evolution [14, 17, 18, 114, 117, 118]. We analyzed the expression of organellar genes and their norgDNAs as a proxy for function, finding that those organelle-derived genes were generally expressed at a far lower level than their organellar counterparts, suggesting limited functional potential. Therefore, while expression of norgDNA does appear to occur, it may reflect leaky transcription rather than function. Expressed and potentially functional norgDNAs, however, do occur in plants (despite the lack of a nuclear promoter) [11], cautioning against a ubiquitous assumption of non-function. Additionally, as we noted, characterization of norgDNA expression needs to acknowledge the high similarity of organelle-derived reads to those from nuclear integrants.

### Evolutionary asymmetry of norgDNAs in the allopolyploidization process of *Gossypium*

During polyploid evolution, many of the *numts* present in the diploids have been lost and nearly all *nupts* in diploids are still retained in current polyploids. Given that assembly quality differences among the genomes represents a possible alternative explanation for presence vs. absence of specific genes, we compared our results to a new analysis using the more recently released and even higher quality genome assemblies for *G. hirsutum* and *G. barbadense* [86]. This analysis mostly reiterated our results, but with some differences. In *G. hirsutum*, there are three additional *numts* (*sdh3*, *rpl5* and *rps7*) and one lost *numt* (*nad4*), and one intact *nupt* (*matK,* the previous inference was not intact) and one decayed *nupt* (*rps12*, the previous inference was intact). In *G. barbadense*, there are six new *numts* (*sdh4*, *cob*, *cox1*, *rpl5*, *rps7* and *rps14*) and four lost *numts* (*nad3*, *nad4L*, *rps12* and *mttB*), and four newly decayed *nupts* (*ycf2, ycf3, ycf4* and *rps12*) that were intact using the earlier released assembly. These results emphasize both the potential impact of assembly quality on inference of *numt* gain and loss, but also confirm that our general conclusions about differential loss and gain in the polyploids are valid. In addition, we also detected some previously published large-fragment transfers, such as the complete mitochondrial genome transfers in Chr01 of *G. raimondii* and ChrA03 of *G. hirsutum* [64, 93], the *numt* on Chr2 of *A. thaliana* [61, 101], and additional, newly detected transfers, further validating reliability and robustness of our methods*.* Therefore, we conclude that the *numt* and *nupt* dynamism reported here is a genuine biological phenomenon.

## Conclusions

This study has concluded that among the three intracellular genomes, most IGT was from organelles into the nuclear genome in two cultivated allopolyploids and their ancestral diploid cotton species; however, both nuclear retrotransposons and chloroplast tRNA genes integrated into mitochondrial genomes at a rate sufficient to correlate with mitogenome size increase. We detected hotspot regions for both the source of IGT (e.g., the *atp6-trnW* intergenic region) and the destination, which require further study in diverse plants to determine the patterns and generality of these observations. We also found that following allopolyploidy, there was a striking asymmetry in IGT retention in the nuclear genome, with most *numts* being lost but most *nupts* retained*.* While it is tempting to attribute parental origin to the loss of these fragments, in that paternally-derived norgDNAs could potentially be interfering, and therefore deleterious, we saw no bias in loss with respect to parental origin. As this is the first report of the relationship between intergenomic gene transfer and allotetraploidy, data from additional polyploid systems is required to understand the evolutionary dynamics of IGT in polyploids.

## Methods

### Plant materials and genome data

We used two varieties of upland cotton (*G. hirsutum*), Xinluzao 11 (X11) and Xinluzhong 42 (X42) for expression analysis. The seeds of X11 and X42 were provided by our own laboratory. X11 (original name: Yuzao 202) was introduced by Bole Seed Station from the Institute of Cash Crops, Henan Academy of Agricultural Sciences, Henan, China, in 1994. After many years of breeding trial, it was approved by the Crop Variety Approval Committee of Xinjiang Autonomous Region, Xinjiang, China, in 1999, and named as Xinluzao 11. X42 was bred by the Institute of Cash Crops of Xinjiang Academy of Agricultural Sciences and approved by the Crop Variety Approval Committee of Xinjiang Autonomous Region, Xinjiang, China, in 2009.

The chloroplast, mitochondrial and nuclear genome sequences of two diploids (*G. raimondii* and *G. arboreum*), and two allotetraploids (*G. hirsutum* and *G. barbadense*) were downloaded from the NCBI database (accession numbers listed in Additional file 1).

### Identification of intergenomic-transfer gene

For each species in the study, we performed pair-wise comparisons of chloroplast or mitochondrial genes and nuclear chromosomes sequences using BLAST (command code 1 in Additional file 9) [118]. We set *e*-value to 1e^− 5^ and a 100-bp minimal length for a high match (95%). We also identified the mitochondrial insertions of chloroplast DNA (*mtpts*) using local BLASTN (version 2.2.23) with the 50-bp minimal length of the match (identity > 95%, coverage > 90%). We cataloged those transfers as full-length genes. Pseudogenes are without full-length or existing mutations resulting in premature stop codons.

### Detection of nuclear transposable elements and repeats in mitochondrial genomes

We detected nuclear transposable elements from nuclear sources using RepeatMasker (command code 2 in Additional file 9) (http://www.repeatmasker.org) with a custom *Gossypium*-enriched repeat database for the four cotton species studied. We used two-tailed *t*-tests to evaluate the significant levels of the different types. The repeats in mitochondrial genome were identified by repeat-match algorithm (command code 3 in Additional file 9) in MUMmer [116]. Specific parameters include: -f (use the forward strand only), −n (minimum match length; default 20), and -t (only output tandem repeats).

### Microhomologies analysis

The analysis was performed as previously described [13, 119]. If there were same nucleotides next to the *mtpt* fusion point shared by the different land species, we identified as microhomologies.

### IGT hotspot analyses in *Gossypium*

We performed dot matrix comparisons between the mitochondrial or chloroplast genomes and nuclear chromosomes of four *Gossypium* species using nucmer program of MUMmer. We set 100-bp minimum size for an exact match and 500-bp minimal interval between every two matches [116]. We calculated middle positions of all *Gossypium* organellar insertions into nuclear chromosomes to tabulate transfer hotspots. Then, we draw the charts of the frequency distribution by R (command code 4 in Additional file 9) (https://www.r-project.org/).

### Expression analysis of norgDNAs

Extracted total RNA using improved cetyltrimethylammonium bromide (CTAB) and sodium dodecyl sulfate (SDS) method from leaves of two accessions of upland cotton, X11 and X42, were sequenced on an Illumina HiSeq2500 at Shanghai Hanyu Biotech Co., Ltd. Sequencing libraries were generated using the Illumina TruSeq RNA Sample Preparation Kit (Illumina, USA) following the manufacturer’s recommendations, and four index codes were added to diagnose the sample origins (nuclear or organellar) for each sequence. Following experimental confirmation of concentration and purity, poly-(T) oligo-attached magnetic beads were utilized for nuclear mRNA enrichment. Fragments, preferentially 200-300 bp in length, were enriched using the Illumina PCR Primer Cocktail in a 10 cycle PCR amplification to form cDNA libraries. Finally, libraries were the paired-end sequenced in one lane with 4 Gb clean reads/sample of an average length of 125 nt. RNA sequences data quality was checked using FastQC. The reads were mapped to the norgDNAs homologies using bowtie 2 (command code 5 in Additional file 7) [120], then samtools idxstats [121] (command code 6 in Additional file 9) were used to calculate the expression reads counts of each gene. The RPKM values were used to estimate relative expressions. Expressed paired-end reads were mapped onto their respective consensus sequences using BWA 0.7.10- r789 [122]; then the results were transformed into BAM files using SAMtools view [121]; and structural variations (SVs) and InDels were visualized using the Integrative Genomics Viewer [123]. The total RNA of X42 and X11 were reverse-transcribed using two pairs of primers at once, i.e., oligo dT primer and random 6 mers, to capture both nuclear expression (NE) and organellar expression (OE). The cDNA produced by the oligo dT primer represents nuclear expression, whereas cDNA produced by the random 6 mers represents a combination of nuclear and organellar genes (NOE). OE is equal to NOE minus NE. Both kinds of cDNA were used as the templates for qRT-PCR. qRT-PCR experiments were conducted using SYBR Premix Ex Taq™ (Tli RNaseH Plus) RR420A kit (TaKaRa) by Applied Biosystems 7500 Real-Time PCR System. The procedure consisted of three stages: stage 1, 95 °C, 30 s, 1 cycle; stage 2: 95 °C, 5 s, 60 °C, 35 s, 40 cycles; stage 3: 95 °C, 15 s, 60 °C, 1 min, 95 °C, 35 s, 1 cycle. Using the cotton housekeeping gene *UBQ7* as internal control, we analysed the relative expression levels of two organellar genes and their nuclear copies, using the 2^−△△Ct^ method. Each sample is repeated three times.

## Supplementary information

**Additional file 1.** Accession numbers for nuclear, mitochondrial and chloroplast genomes of four cotton species.

**Additional file 2.** Correlation between the length of nuclear and chloroplast sequences transferring to mitochondrial genome, mitochondrial genome size and repeat sizes in mitochondrial genomes in 26 land plants. (A) Correlation between the length of the nuclear sequences transferring to the mitochondrion and mitochondrial genome size. (B) Correlation between the length of the chloroplast sequences transferring to the mitochondrion and mitochondrial genome size. (C) Correlation between repeat sizes in mitochondrial genomes and the mitochondrial genome. (D) Correlation between length of nuclear sequences transferring to mitochondrial genomes and repeat sizes of mitochondrial genomes. (E) Correlation between length of chloroplast sequences transferring to mitochondrial genomes and repeat sizes of mitochondrial genomes. Each dot represents a two-dimensional value (X, Y) of one species. Back dots denote four cotton species and the gray dots mean the other species. The slash represents the linear regression function of the distribution tendency of the dots. *R*^2^ is the regression coefficient.

**Additional file 3.** Dot matrix analysis of nuclear insertions of chloroplast DNAs (above) and mitochondrial DNAs (below) in 14 plant species including four cotton species identified by whole-genome alignment. The results were filtered so that only those alignments with the one-to-one mapping between reference and query were selected. The red and blue lines refer to positive and reverse matches, respectively. The uppermost phylogenetic clades are drawn based on the maximum likelihood (ML) method with the model GTR + G + I. The figures related to mitochondrial DNAs insertions into four the nuclear genomes of four cotton species are quoted from a previous paper in our lab [61].

**Additional file 4.** The identification of nuclear organellar DNA in *Gossypium*. A: *G. raimondii*. B: *G. arboreum*. C: *G. hirsutum* (A_*t*_). D: *G. hirsutum* (D_*t*_). Red bands around the circles indicate the nuclear chromosomes. Orange and green lines represent insertions more than 5 kb from mitogenome and chloroplast genome, respectively. While grey lines represent insertions between 100 bp to 5 kb from both genomes. MT: mitochondrial genome. CP: chloroplast genome.

**Additional file 5.** Correlation between the length of mitochondrial and chloroplast sequences transferring to the nuclear genome, nuclear genome size and repeat sizes in nuclear genomes in 26 land plants. (A) Correlation between the length of mitochondrial sequences transferring to nuclear and nuclear genome sizes. (B) Correlation between the length of the chloroplast sequences transferring to nuclear and nuclear genome sizes. (C) Correlation between repeat sizes in nuclear genomes and nuclear genome sizes. (D) Correlation between length of mitochondrial sequences transferring to nuclear genomes and repeat sizes of nuclear genomes. (E) Correlation between length of chloroplast sequences transferring to nuclear genomes and repeat sizes of nuclear genomes. Each dot represents a two-dimensional value (X, Y) of one species. Black dots denote four cotton species and the gray dots mean the other species. The slash represents the linear regression function of the distribution tendency of the dots. *R*^2^ is the regression coefficient.

**Additional file 6.** RNA-seq expressed paired-end reads of chloroplast gene *petG*_cp and its nuclear homology *petG*_D12 in *G. hirsutum* variety (Xinluzao 11). The two images above show the variability and coverage of the RNA-seq expressed paired-end reads via an Integrative Genomics Viewer (IGV) screenshot. Each image contains three main panels. The upper panel represents the sequence coordinates. The middle panel is subdivided into two tracks, where the upper track depicts read density and the lower track shows the mapping of clean reads. The panel at the bottom represents the linear DNA sequence. The SNPs in *petG*_D12 and the corresponding normal nucleotide acids in *petG*_cp are highlighted in bold. The expressed reads of *petG*_D12 share the same nucleotide acids sequences with *petG*_cp and few expressed reads are mapped to the divergence region.

**Additional file 7.** Relative expression levels of chloroplast genes *atpE_cp/petG_cp* and their nuclear copies, *atpE_A09/petG_D12*, in two *G. hirsutum* varieties. X42: Xinluzao 42; X11: Xinluzao 11. The relative expression values are calculated with the method of 2^−△△Ct^. **, *p* < 0.01. See Methods for details.

**Additional file 8.** Hypothetic evolutionary model showing the change of norgDNAs during allopolyploidization of *Brassica* species. (A) Schema graph showing the mitochondrial-to-nuclear IGT events during the allopolyploidy of *Brassica*. CC: *B. oleracea.* AA: *B. rapa*. AACC: *B. napus*. Gray rectangular strips represent gene blocks that transferred before the divergence of two diploid species. Blue and purple genes strips represent gene blocks that transferred only in diploid CC and allotetraploid AACC, respectively. Genes in green color denote pseudogenes. (B) Schema graph showing the chloroplast-to-nuclear IGT events during the allopolyploidy of *Brassica*. Genes in the same gray boxes belong to one functional classification. The genes in green color denote pseudogenes. Genes in blue, purple and red boxes transferred only in CC, AA, and AACC, respectively.

**Additional file 9.** Commands and their codes used in this study.

## Data Availability

The datasets used and/or analysed during the current study are available in the NCBI repository. *G. raimondii* nuclear genome, accession number: PRJNA171262, DOI: https://www.ncbi.nlm.nih.gov/bioproject/PRJNA171262; *G. raimondii* mitochondrial genome, accession number: KR736345, DOI: https://www.ncbi.nlm.nih.gov/nuccore/KR736345.1/; *G. raimondii* chloroplast genome, accession number: HQ325744, DOI: https://www.ncbi.nlm.nih.gov/nuccore/HQ325744.1/; *G. arboreum* nuclear genome, accession number: PRJNA335838, DOI: https://www.ncbi.nlm.nih.gov/bioproject/PRJNA335838; *G. arboreum* mitochondrial genome, accession number: KR736342, DOI: https://www.ncbi.nlm.nih.gov/nuccore/KR736342.1/; *G. arboreum* chloroplast genome, accession number: HQ325740, DOI: https://www.ncbi.nlm.nih.gov/nuccore/HQ325740.1/; *G. hirsutum* nuclear genome, accession number: PRJNA248163, DOI: https://www.ncbi.nlm.nih.gov/bioproject/PRJNA248163; *G. hirsutum* mitochondrial genome, accession number: JX944505, DOI: https://www.ncbi.nlm.nih.gov/nuccore/JX944505.1/; *G. hirsutum* chloroplast genome, accession number: DQ345959, DOI: https://www.ncbi.nlm.nih.gov/nuccore/DQ345959.1/; *G. barbadense* nuclear genome, accession number: PRJNA219156, DOI: https://www.ncbi.nlm.nih.gov/bioproject/PRJNA219156; *G. barbadense* mitochondrial genome, accession number: KP898249, DOI: https://www.ncbi.nlm.nih.gov/nuccore/KP898249.1/; *G. barbadense* chloroplast genome, accession number: AP009123, DOI: https://www.ncbi.nlm.nih.gov/nuccore/AP009123.1/; Other datasets supporting the conclusions of this article are included within the article and its additional files.

## Abbreviations

*B. napus* (AACC): *Brassica napus*
*B. oleracea* (CC): *Brassica oleracea*
*B. rapa* (AA): *Brassica rapa*
cpDNA: Chloroplast DNA
*G. arboreum*,(A_2_): *Gossypium arboreum*
*G. barbadense* (AD_2_): *barbadense*
*G. herbaceum* (A_1_): *Gossypium herbaceum*
*G. hirsutum* (AD_1_): *Gossypium hirsutum*
*G. raimondii* (D_5_): *Gossypium raimondii*
IGT: Intergenomic gene transfer
LTR-retro: Long terminal repeat retrotransposon
mtDNA: Mitochondrial DNA
norgDNAs: Nuclear organellar DNAs
nuDNA: Nuclear DNA
*numts*: Nuclear mitochondrial insertions
nupts: Nuclear plastidial insertions
TE: Transposable element
