# Supplementary material for: Intergenomic gene transfer in diploid and allopolyploid Gossypium
Source: BMC Plant Biol. 2019 Nov 12;19:492. doi: 10.1186/s12870-019-2041-2 (PMC6852956; doi:10.1186/s12870-019-2041-2)
Supplement: Supplementary file 2 — Additional file 2. Correlation between the length of nuclear and chloroplast sequences transferring to mitochondrial genome, mitochondrial genome size and repeat sizes in mitochondrial genomes in 26 land plants. (A) Correlation between the length of the nuclear sequences transferring to the mitochondrion and mitochondrial genome size. (B) Correlation between the length of the chloroplast sequences transferring to the mitochondrion and mitochondrial genome size. (C) Correlation between repeat sizes in mitochondrial genomes and the mitochondrial genome. (D) Correlation between length of nuclear sequences transferring to mitochondrial genomes and repeat sizes of mitochondrial genomes. (E) Correlation between length of chloroplast sequences transferring to mitochondrial genomes and repeat sizes of mitochondrial genomes. Each dot represents a two-dimensional value (X, Y) of one species. Back dots denote four cotton species and the gray dots mean the other species. The slash represents the linear regression function of the distribution tendency of the dots. R2 is the regression coefficient. [file 12870_2019_2041_MOESM2_ESM.docx]

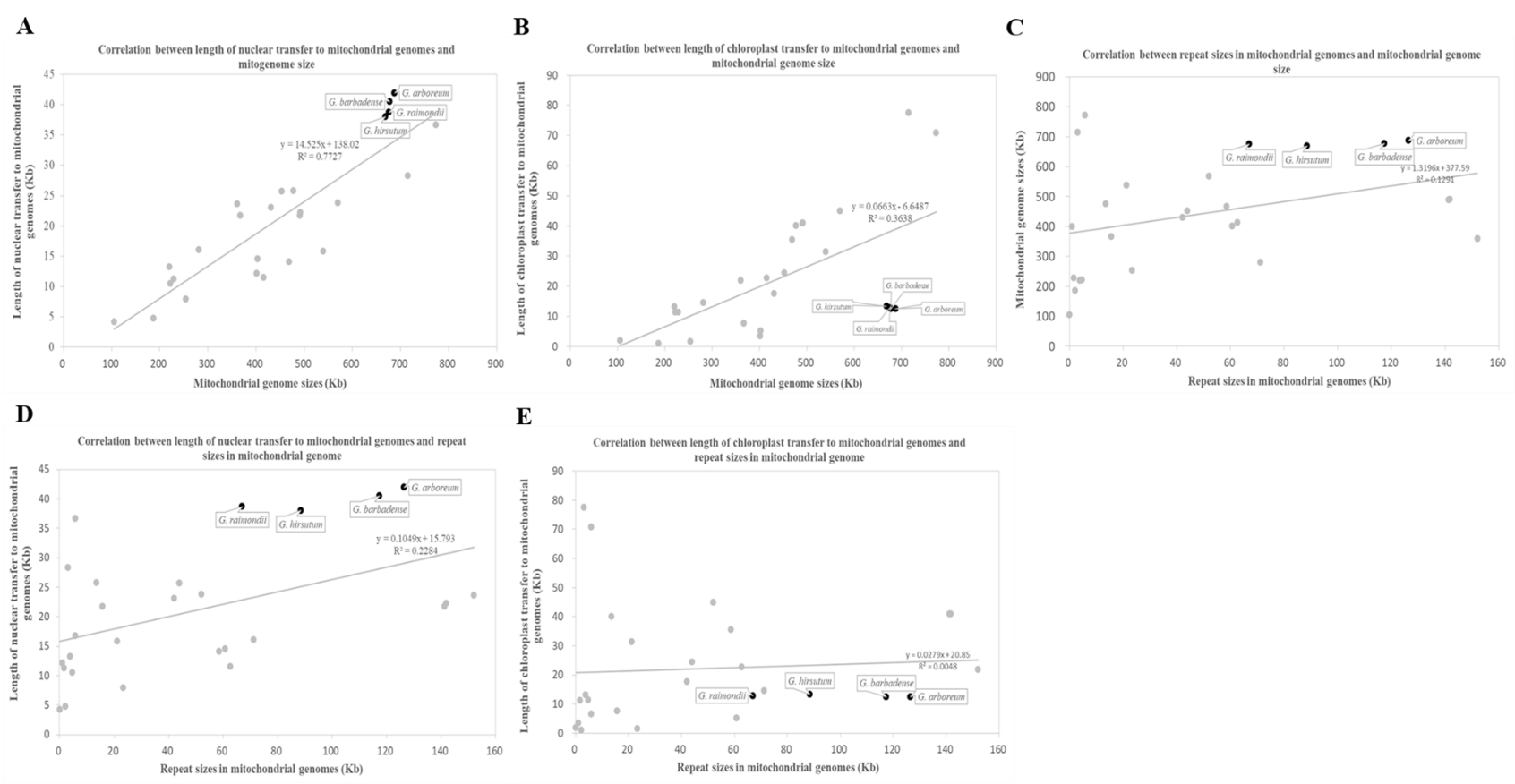


**Additional file 2:** Correlation between the length of nuclear and chloroplast sequences transferring to mitochondrial genome, mitochondrial genome size and repeat sizes in mitochondrial genomes in 26 land plants. (A) Correlation between the length of the nuclear sequences transferring to the mitochondrion and mitochondrial genome size. (B) Correlation between the length of the chloroplast sequences transferring to the mitochondrion and mitochondrial genome size. (C) Correlation between repeat sizes in mitochondrial genomes and the mitochondrial genome. (D) Correlation between length of nuclear sequences transferring to mitochondrial genomes and repeat sizes of mitochondrial genomes. (E) Correlation between length of chloroplast sequences transferring to mitochondrial genomes and repeat sizes of mitochondrial genomes. Each dot represents a two-dimensional value (X, Y) of one species. Back dots denote four cotton species and the gray dots mean the other species. The slash represents the linear regression function of the distribution tendency of the dots. R^2^ is the regression coefﬁcient.
