# Supplementary material for: Intergenomic gene transfer in diploid and allopolyploid Gossypium
Source: BMC Plant Biol. 2019 Nov 12;19:492. doi: 10.1186/s12870-019-2041-2 (PMC6852956; doi:10.1186/s12870-019-2041-2)
Supplement: Supplementary file 3 — Additional file 3. Dot matrix analysis of nuclear insertions of chloroplast DNAs (above) and mitochondrial DNAs (below) in 14 plant species including four cotton species identified by whole-genome alignment. The results were filtered so that only those alignments with the one-to-one mapping between reference and query were selected. The red and blue lines refer to positive and reverse matches, respectively. The uppermost phylogenetic clades are drawn based on the maximum likelihood (ML) method with the model GTR + G + I. The figures related to mitochondrial DNAs insertions into four the nuclear genomes of four cotton species are quoted from a previous paper in our lab [61]. [file 12870_2019_2041_MOESM3_ESM.docx]

**
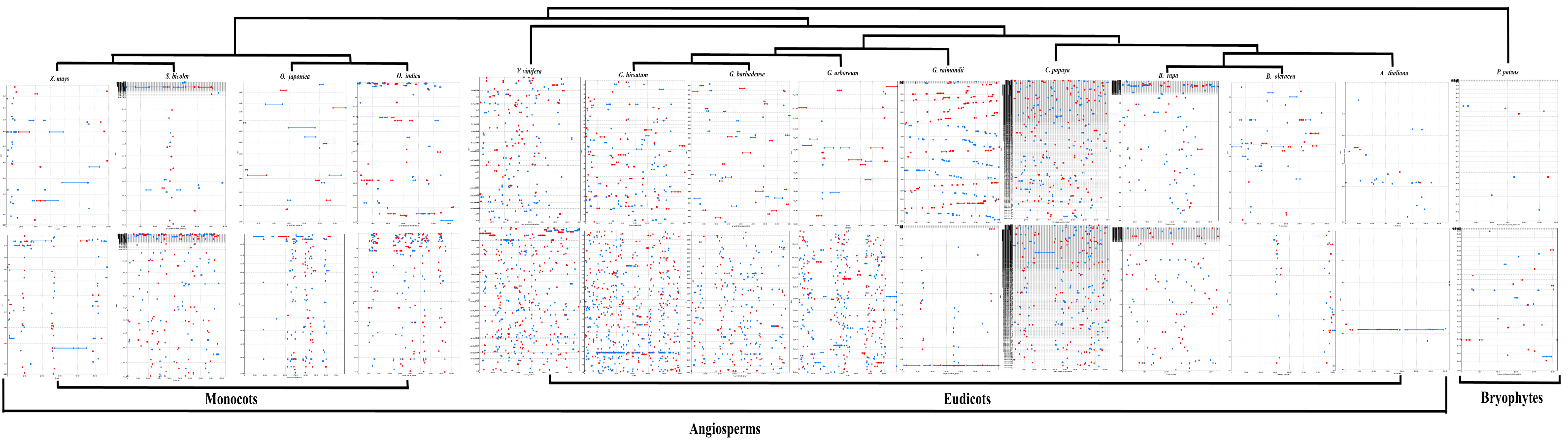
**

**Additional file 3:** Dot matrix analysis of nuclear insertions of chloroplast DNAs (above) and mitochondrial DNAs (below) in 14 plant species including four cotton species identified by whole-genome alignment. The results were filtered so that only those alignments with the one-to-one mapping between reference and query were selected. The red and blue lines refer to positive and reverse matches, respectively. The uppermost phylogenetic clades are drawn based on the maximum likelihood (ML) method with the model GTR + G + I. The figures related to mitochondrial DNAs insertions into four the nuclear genomes of four cotton species are quoted from a previous paper in our lab [61].
