# Supplementary material for: Intergenomic gene transfer in diploid and allopolyploid Gossypium
Source: BMC Plant Biol. 2019 Nov 12;19:492. doi: 10.1186/s12870-019-2041-2 (PMC6852956; doi:10.1186/s12870-019-2041-2)
Supplement: Supplementary file 4 — Additional file 4. The identification of nuclear organellar DNA in Gossypium. A: G. raimondii. B: G. arboreum. C: G. hirsutum (At). D: G. hirsutum (Dt). Red bands around the circles indicate the nuclear chromosomes. Orange and green lines represent insertions more than 5 kb from mitogenome and chloroplast genome, respectively. While grey lines represent insertions between 100 bp to 5 kb from both genomes. MT: mitochondrial genome. CP: chloroplast genome. [file 12870_2019_2041_MOESM4_ESM.docx]

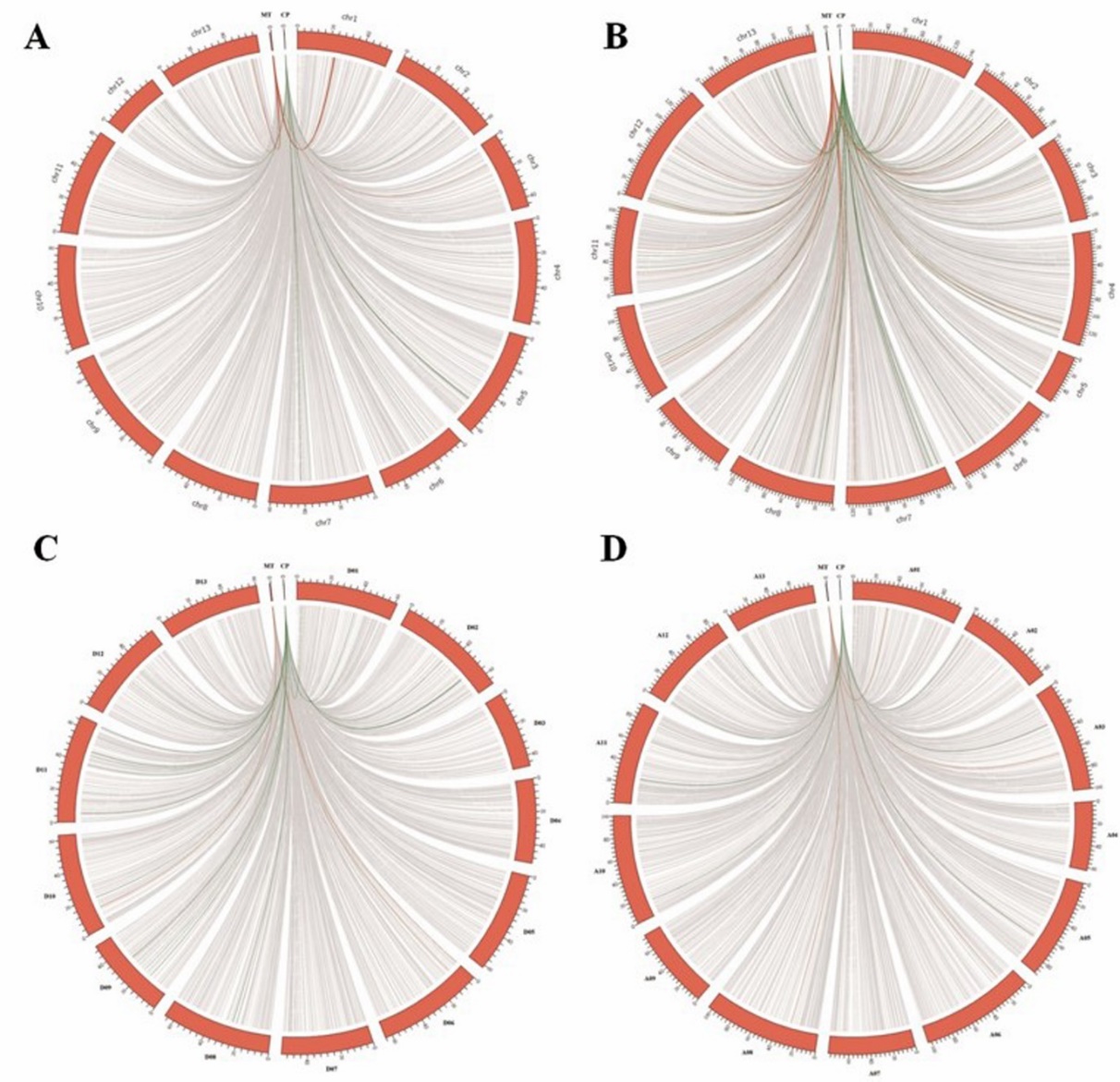


**Additional file 4：**The identification of nuclear organellar DNA in *Gossypium*. A: *G. raimondii*. B: *G. arboreum*. C: *G. hirsutum* (A*_t_*). D: *G. hirsutum* (D*_t_*). Red bands around the circles indicate the nuclear chromosomes. Orange and green lines represent insertions more than 5 kb from mitogenome and chloroplast genome, respectively. While grey lines represent insertions between 100bp to 5 kb from both genomes. MT: mitochondrial genome. CP: chloroplast genome.
