# Supplementary material for: Intergenomic gene transfer in diploid and allopolyploid Gossypium
Source: BMC Plant Biol. 2019 Nov 12;19:492. doi: 10.1186/s12870-019-2041-2 (PMC6852956; doi:10.1186/s12870-019-2041-2)
Supplement: Supplementary file 5 — Additional file 5. Correlation between the length of mitochondrial and chloroplast sequences transferring to the nuclear genome, nuclear genome size and repeat sizes in nuclear genomes in 26 land plants. (A) Correlation between the length of mitochondrial sequences transferring to nuclear and nuclear genome sizes. (B) Correlation between the length of the chloroplast sequences transferring to nuclear and nuclear genome sizes. (C) Correlation between repeat sizes in nuclear genomes and nuclear genome sizes. (D) Correlation between length of mitochondrial sequences transferring to nuclear genomes and repeat sizes of nuclear genomes. (E) Correlation between length of chloroplast sequences transferring to nuclear genomes and repeat sizes of nuclear genomes. Each dot represents a two-dimensional value (X, Y) of one species. Black dots denote four cotton species and the gray dots mean the other species. The slash represents the linear regression function of the distribution tendency of the dots. R2 is the regression coefficient. [file 12870_2019_2041_MOESM5_ESM.docx]

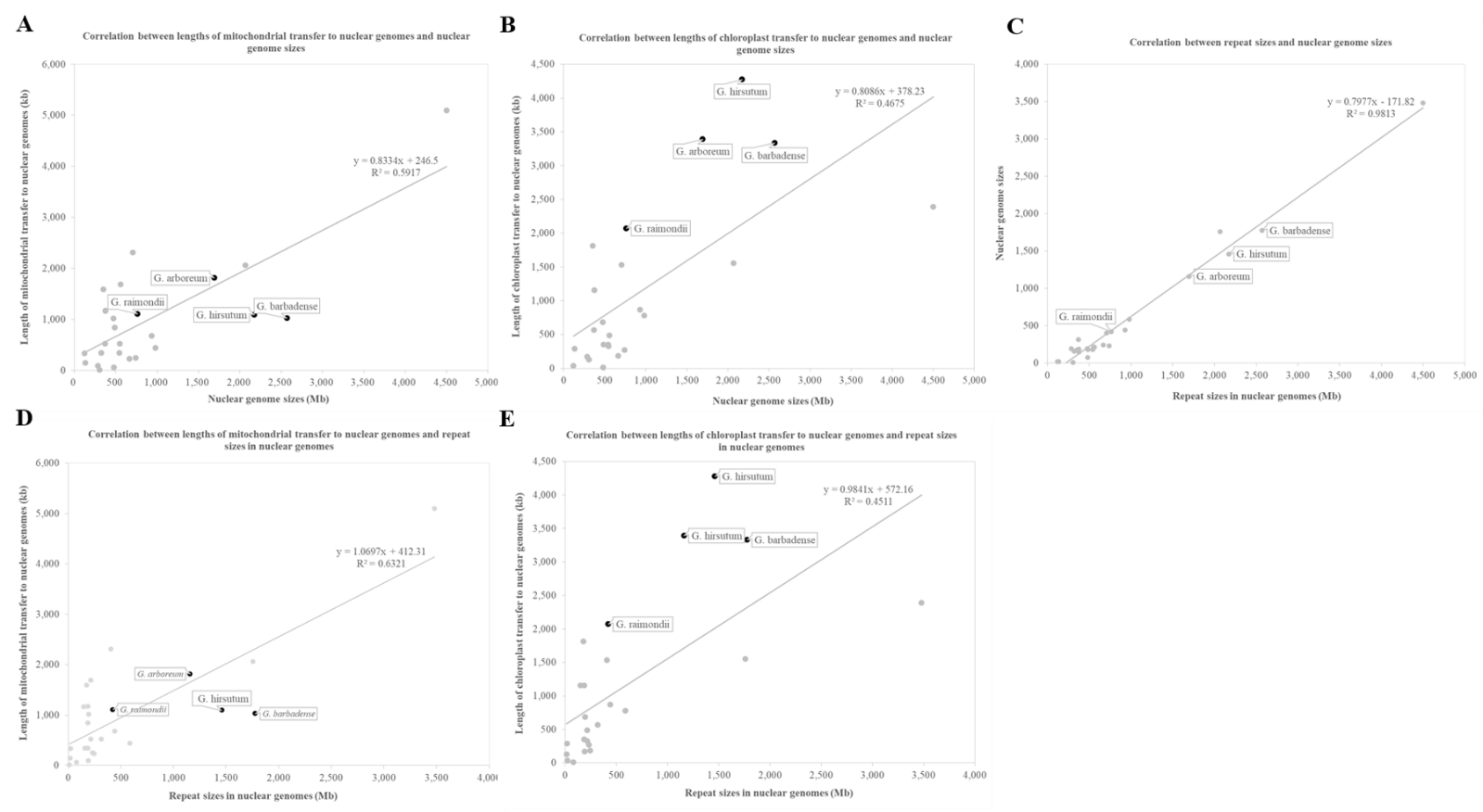


**Additional file 5:** Correlation between the length of mitochondrial and chloroplast sequences transferring to the nuclear genome, nuclear genome size and repeat sizes in nuclear genomes in 26 land plants. (A) Correlation between the length of mitochondrial sequences transferring to nuclear and nuclear genome sizes. (B) Correlation between the length of the chloroplast sequences transferring to nuclear and nuclear genome sizes. (C) Correlation between repeat sizes in nuclear genomes and nuclear genome sizes. (D) Correlation between length of mitochondrial sequences transferring to nuclear genomes and repeat sizes of nuclear genomes. (E) Correlation between length of chloroplast sequences transferring to nuclear genomes and repeat sizes of nuclear genomes. Each dot represents a two-dimensional value (X, Y) of one species. Black dots denote four cotton species and the gray dots mean the other species. The slash represents the linear regression function of the distribution tendency of the dots. R^2^ is the regression coefﬁcient.
