# Supplementary material for: Intergenomic gene transfer in diploid and allopolyploid Gossypium
Source: BMC Plant Biol. 2019 Nov 12;19:492. doi: 10.1186/s12870-019-2041-2 (PMC6852956; doi:10.1186/s12870-019-2041-2)
Supplement: Supplementary file 6 — Additional file 6. RNA-seq expressed paired-end reads of chloroplast gene petG_cp and its nuclear homology petG_D12 in G. hirsutum variety (Xinluzao 11). The two images above show the variability and coverage of the RNA-seq expressed paired-end reads via an Integrative Genomics Viewer (IGV) screenshot. Each image contains three main panels. The upper panel represents the sequence coordinates. The middle panel is subdivided into two tracks, where the upper track depicts read density and the lower track shows the mapping of clean reads. The panel at the bottom represents the linear DNA sequence. The SNPs in petG_D12 and the corresponding normal nucleotide acids in petG_cp are highlighted in bold. The expressed reads of petG_D12 share the same nucleotide acids sequences with petG_cp and few expressed reads are mapped to the divergence region. [file 12870_2019_2041_MOESM6_ESM.docx]

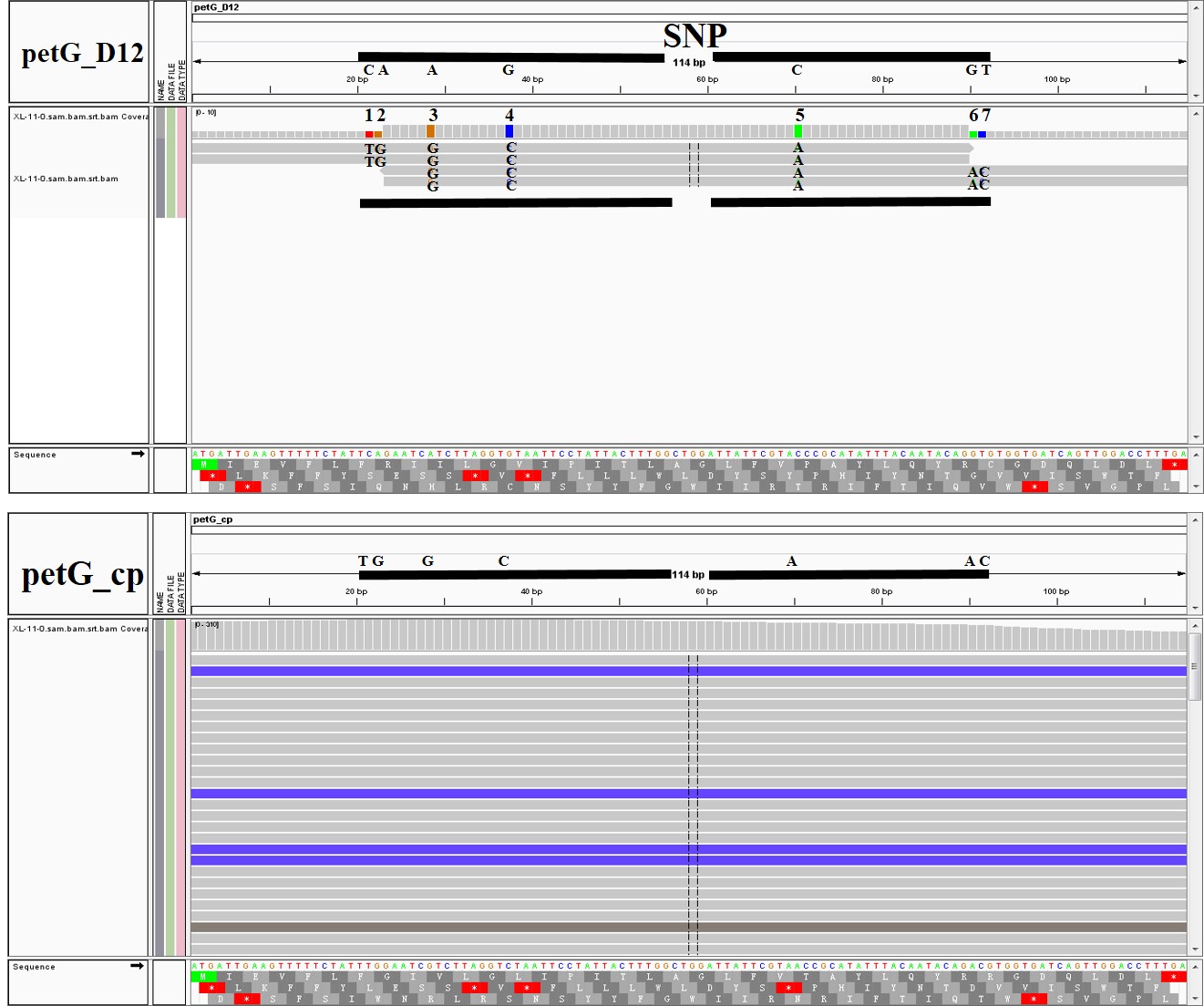


**Additional file 6:** RNA-seq expressed paired-end reads of chloroplast gene *petG*_cp and its nuclear homology *petG*_D12 in *G. hirsutum* variety (Xinluzao 11). The two images above show the variability and coverage of the RNA-seq expressed paired-end reads via an Integrative Genomics Viewer (IGV) screenshot. Each image contains three main panels. The upper panel represents the sequence coordinates. The middle panel is subdivided into two tracks, where the upper track depicts read density and the lower track shows the mapping of clean reads. The panel at the bottom represents the linear DNA sequence. The SNPs in *petG*_D12 and the corresponding normal nucleotide acids in *petG*_cp are highlighted in bold. The expressed reads of *petG*_D12 share the same nucleotide acids sequences with *petG*_cp and few expressed reads are mapped to the divergence region.
