# Supplementary material for: Intergenomic gene transfer in diploid and allopolyploid Gossypium
Source: BMC Plant Biol. 2019 Nov 12;19:492. doi: 10.1186/s12870-019-2041-2 (PMC6852956; doi:10.1186/s12870-019-2041-2)
Supplement: Supplementary file 7 — Additional file 7. Relative expression levels of chloroplast genes atpE_cp/petG_cp and their nuclear copies, atpE_A09/petG_D12, in two G. hirsutum varieties. X42: Xinluzao 42; X11: Xinluzao 11. The relative expression values are calculated with the method of 2−△△Ct. **, p < 0.01. See Methods for details. [file 12870_2019_2041_MOESM7_ESM.docx]

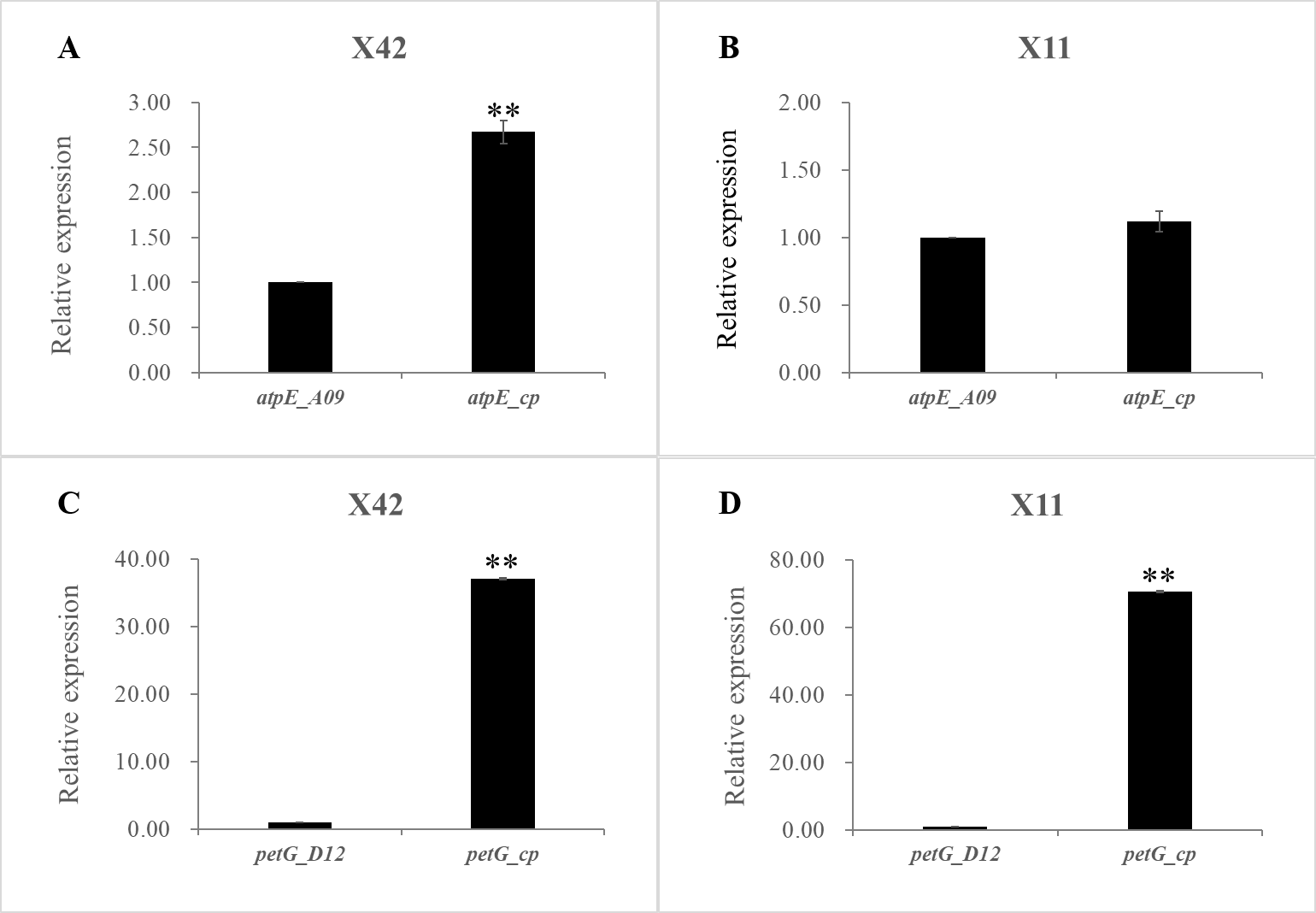


**Additional file 7:** Relative expression levels of chloroplast genes *atpE_cp/petG_cp* and their nuclear copies, *atpE_A09/petG_D12*, in two *G. hirsutum* varieties. X42: Xinluzao 42; X11: Xinluzao 11. The relative expression values are calculated with the method of 2^−△△Ct^. **, p<0.01. See methods for details.
