# Supplementary material for: Intergenomic gene transfer in diploid and allopolyploid Gossypium
Source: BMC Plant Biol. 2019 Nov 12;19:492. doi: 10.1186/s12870-019-2041-2 (PMC6852956; doi:10.1186/s12870-019-2041-2)
Supplement: Supplementary file 8 — Additional file 8. Hypothetic evolutionary model showing the change of norgDNAs during allopolyploidization of Brassica species. (A) Schema graph showing the mitochondrial-to-nuclear IGT events during the allopolyploidy of Brassica. CC: B. oleracea. AA: B. rapa. AACC: B. napus. Gray rectangular strips represent gene blocks that transferred before the divergence of two diploid species. Blue and purple genes strips represent gene blocks that transferred only in diploid CC and allotetraploid AACC, respectively. Genes in green color denote pseudogenes. (B) Schema graph showing the chloroplast-to-nuclear IGT events during the allopolyploidy of Brassica. Genes in the same gray boxes belong to one functional classification. The genes in green color denote pseudogenes. Genes in blue, purple and red boxes transferred only in CC, AA, and AACC, respectively. [file 12870_2019_2041_MOESM8_ESM.docx]

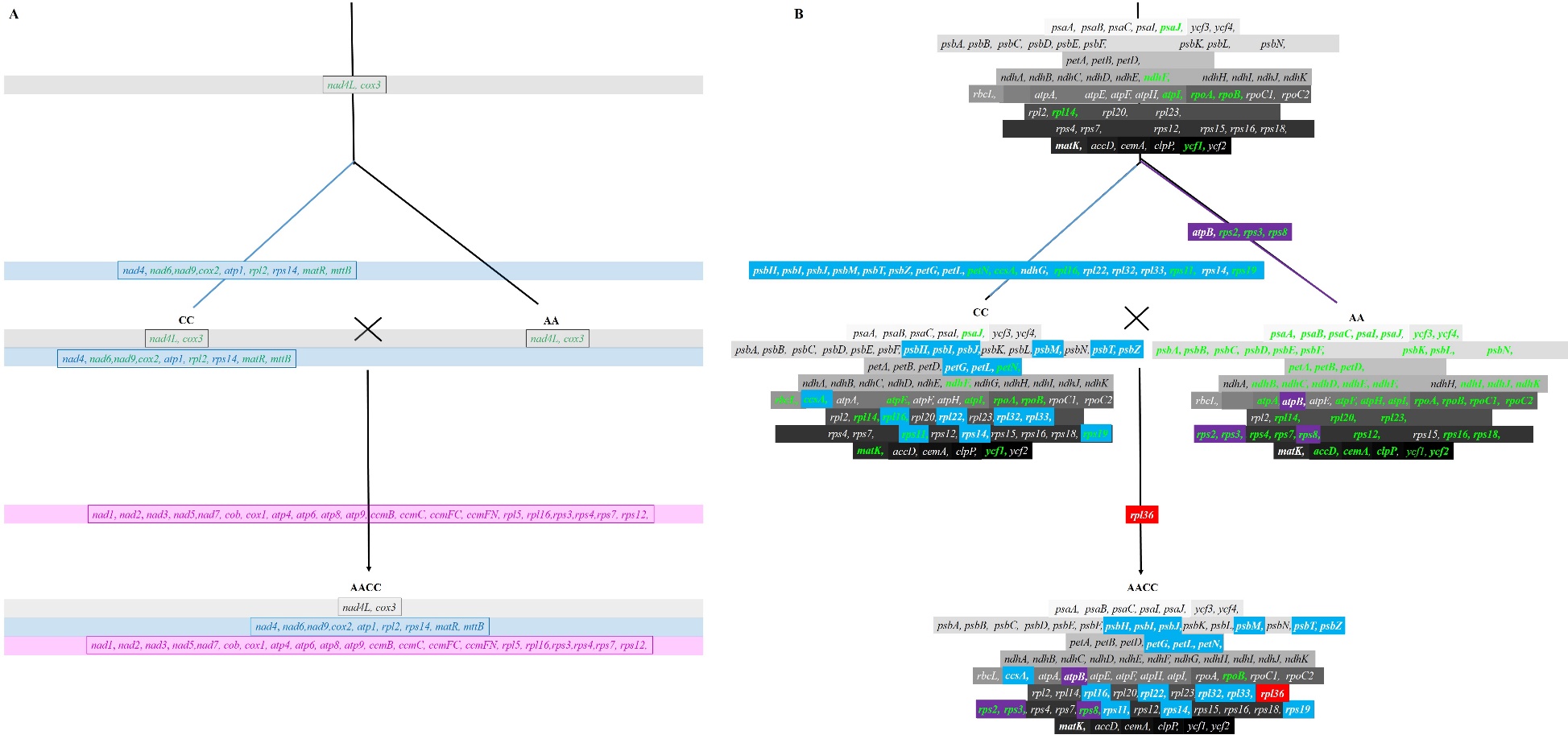


**Additional file 8:** Hypothetic evolutionary model showing the change of norgDNAs during allopolyploidization of *Brassica* species. (A) Schema graph showing the mitochondrial-to-nuclear IGT events during the allopolyploidy of *Brassica*. CC: *B. oleracea.* AA: *B. rapa*. AACC: *B. napus*. Gray rectangular strips represent gene blocks that transferred before the divergence of two diploid species. Blue and purple genes strips represent gene blocks that transferred only in diploid CC and allotetraploid AACC, respectively. Genes in green color denote pseudogenes. (B) Schema graph showing the chloroplast-to-nuclear IGT events during the allopolyploidy of *Brassica*. Genes in the same gray boxes belong to one functional classification. The genes in green color denote pseudogenes. Genes in blue, purple and red boxes transferred only in CC, AA, and AACC, respectively.
